# Motor phenotypes and neurofilament light chain in genetic amyotrophic lateral sclerosis—results from a multicenter screening program

**DOI:** 10.1007/s00415-025-13555-6

**Published:** 2025-12-12

**Authors:** Philipp Schmitt, Peggy Schumann, Alexander Koerbs, Hsuen-Ju Lin, Torsten Grehl, Ute Weyen, Susanne Petri, Annekathrin Rödiger, Robert Steinbach, Julian Großkreutz, Sarah Bernsen, Patrick Weydt, Joachim Wolf, René Günther, Petra Baum, Moritz Metelmann, Jochen H. Weishaupt, Berthold Streubel, David C. Kasper, Yasemin Koc, Dagmar Kettemann, Jenny Norden, Bertram Walter, Christoph Münch, Susanne Spittel, André Maier, Péter Körtvélyessy, Thomas Meyer

**Affiliations:** 1 Department of Neurology, Center for ALS and Other Motor Neuron Disorders, Charité-Universitätsmedizin Berlin, Corporate Member of Freie Universität Berlin, Humboldt-Universität Zu Berlin, and Berlin Institute of Health, Augustenburger Platz 1, 13353 Berlin, Germany; 2 Ambulanzpartner Soziotechnologie APST GmbH, Berlin, Germany; 3 Amedes Genetics, Hannover, Germany; 4 Department of Neurology, Center for ALS and Other Motor Neuron Disorders, Alfried Krupp Krankenhaus, Essen, Germany; 5 Department of Neurology, Center for ALS and Other Motor Neuron Disorders, Berufsgenossenschaftliches Universitätsklinikum Bergmannsheil, Bochum, Germany; 6 Department of Neurology, Hannover Medical School, Hannover, Germany; 7 Department of Neurology, Jena University Hospital, Jena, Germany; 8 ZSE, Zentrum für Seltene Erkrankungen, Jena University Hospital, Jena, Germany; 9 Department of Neurology, Universitätsmedizin Schleswig-Holstein, Campus Lübeck, Lübeck, Germany; 10 Department for Neuromuscular Disorders, Bonn University, Bonn, Germany; 11 DZNE, Deutsches Zentrum für Neurodegenerative Erkrankungen, Research Site Bonn, Bonn, Germany; 12 Department of Neurology, Brüderklinikum Julia Lanz, Diako Mannheim, Mannheim, Germany; 13 Department of Neurology, Technische Universität Dresden, University Hospital Carl Gustav Carus, Dresden, Germany; 14 DZNE, Deutsches Zentrum für Neurodegenerative Erkrankungen, Research Site Dresden, Dresden, Germany; 15 Department of Neurology, University Hospital Leipzig, Leipzig, Germany; 16 Department of Neurology, University of Ulm, Ulm, Germany; 17 Institute for Pathology, Medical University of Wien, Vienna, Austria; 18 ARCHIMED Life Science GmbH, Vienna, Austria

**Keywords:** Genetic ALS, Neurofilament, ALS progression, Phenotype

## Abstract

**Objective:**

In genetic amyotrophic lateral sclerosis (ALS), the clinical phenotypes, disease progression and neurofilament light chain (NfL) levels are incompletely characterized.

**Methods:**

In a total cohort of 1988 ALS patients, a subcohort of genetic ALS linked to *C9orf72* (n = 137)*, SOD1* (n = 54), *TARDBP* (n = 27), and *FUS* (n = 19) was investigated. The phenotypes of onset region, propagation and motor neuron involvement were analyzed according to the OPM classification. Serum NfL (sNfL) was measured and related to ALS progression (ALSPR, monthly change of ALS Functional Rating Scale–Revised). To quantify NfL elevation relative to ALSPR, the logNfL(index), the log-transformed ratio of sNfL to ALSPR was calculated.

**Results:**

*C9orf72*-associated ALS showed frequent bulbar onset (n = 42.6%), higher ALSPR (0.95, SD 0.84), highest NfL (116.3, SD 72.7 pg/mL) and logNfL(index) (5.02, SD 0.88). *SOD1-*ALS had mostly limb onset (n = 96.1%), slower ALSPR (0.57, SD 0.60), high NfL (76.1, SD 61.4 pg/mL) and a comparably high logNfL(index) (4.94, SD 1.03). *FUS*-ALS exhibited mostly limb onset (82.4%), lower motor neuron dysfunction (70.6%), a wide range of faster (22.2%) to slower ALSPR (55.6%), lower NfL (66.2, SD 32.9) and logNfL(4.65, SD 0.9). *TARDBP-*ALS displayed the lowest ALSPR (0.53, SD 0.52), the lowest NfL (43.3, SD 31.8 pg/mL) and the lowest logNfL(index) (4.40, SD 0.7).

**Conclusion:**

In *C9orf72*-ALS, the phenotype and NfL profile are close to typical ALS. The finding of distinct phenotypes and NfL patterns in *SOD1*-, *FUS*- and *TARDBP-*associated ALS underscores the relevance of genetic ALS for prognostic counseling, clinical trial design, treatment expectations and unraveling of pathogenic mechanisms in ALS.

**Supplementary Information:**

The online version contains supplementary material available at 10.1007/s00415-025-13555-6.

## Introduction

Amyotrophic lateral sclerosis (ALS) is a progressive and ultimately fatal neurodegenerative disorder primarily affecting motor neurons [1]. Over the past three decades, genetic findings have reshaped the pathogenetic perception of ALS. More than 60 genes have been implicated in its etiology, with *C9orf72*, *SOD1*, *TARDBP* and *FUS* recognized as the most frequently variants [2]. Notably, such pathogenic mutations have also been detected in patients without a documented family history. The reported frequency of genetic ALS within apparently sporadic ALS cohorts can vary considerably, ranging up to 13% in large-scale genetic screenings [3]. Genotype–phenotype correlations—the association of clinical characteristics with genetic variants—are of clinical relevance in genetic counselling in ALS. Furthermore, it can contribute to the individual prognosis of people with ALS carrying a disease-causing mutation. More recently, the approval of the antisense oligonucleotide (ASO) tofersen in *SOD1*-associated ALS lead to a therapeutic relevance of genetic ALS [4–6]. Also in other genetic ALS, such as *FUS*-ALS, efforts in ASO-based interventions are ongoing (NCT04768972). Consequently, a new era of genetic treatment strategies has begun, making the understanding of the genotype–phenotype correlation of additional relevance.

Previous studies have examined the clinical characteristics associated with ALS-causing mutations [7]. Recently, a large European multicenter study investigated the clinical impact of *C9orf72*, *SOD1*, *FUS* and *TARDBP* variants in ALS. Distinct forms of genetic ALS show earlier onset, distinct onset sites and different progression patterns compared to sporadic cases [8]. Although genotype–phenotype correlations emerge, a systematic assessment of the clinical phenotype remains underreported. Neurofilament light chain (NfL) is reportedly a robust biomarker of disease progression [9]. NfL levels correlate significantly with disease progression as measured by the ALS Functional Rating Scale–Revised (ALSFRS-R) and, most importantly, with patient survival [10]. Additionally, serum NfL (sNfL) correlates with ALS phenotypes, particularly regarding motor neuron involvement and the region of onset or propagation [11]. However, few data on NfL in genetic ALS are available.

To address gaps in the understanding of phenotypes and NfL in genetic ALS, existing data of a large multi-center genetic screening program were analyzed, including a standardized clinical assessment and phenotyping and NfL measurement*.* This approach aimed to (1) investigate the phenotypes in terms of onset region, propagation pattern and the degree of upper and lower motor neuron dysfunction, (2) to determine the ALS progression rate, and (3) to measure serum NfL in genetic ALS associated with *C9orf72*, *SOD1*, *TARDBP* and *FUS* mutations.

## Methods

### Study design

This study is a secondary use of existing data from three multicenter studies, in which clinical, phenotypic and NfL data are collected. Data were obtained from (1) a registry of clinical characteristics, phenotypes, and the standard of care (ClinicalTrials.gov ID NCT05852418), (2) a large-scale longitudinal study on sNfL in ALS, and (3) a multicenter study on genetic variants in ALS (ClinicalTrials.gov ID NCT05852405).

### Studied cohort

Data analysis was performed in patients fulfilling the following criteria: (1) diagnosis of ALS according to the Gold Coast criteria [12]; (2) consent to electronic data capture using the research platform “APST” [13] and (3) written consent to genetic investigation.

### Setting

Data analysis included participants at 11 multidisciplinary ALS centers in Germany between October 2021 and June 2024. Demographic and clinical data were obtained from electronic health records. Rating of the ALS function rating scale-revised (ALSFRS-R) was performed by qualified evaluators at the time of blood sampling for serum NfL analysis. Genetic data were obtained from existing data of an ALS genetic screening program [14].

### NfL analysis

Blood samples were collected and shipped to the ALS center in Berlin (Germany) where the core facility for NfL analysis was located. Measurement of sNfL concentration was performed with the single molecule array (SIMOA) technology (HD-X instrument, Quanterix Inc.) using the commercially available NfL Advantage Kit (Quanterix Inc.). In SOD1-ALS patients receiving tofersen treatment, only NfL values before the initiation of therapy were included.

### Variables

#### Demographic and clinical characteristics

The following demographic and clinical characteristics were collected: age at disease onset, sex, disease duration (number of months between disease onset and study inclusion), and ALSFRS-R [15].

#### Frequency of genetic variants

Genetic variants of the *SOD1*, *FUS* and *TARDBP* genes and pathogenic HRE of C9orf72 were assessed. Detailed information about the genetic variant (substitution, deletion, insertion, duplication inversion) and location were collected. Pathogenicity of genetic variants was classified according to the ACMG guidelines [16], with modifications based on the recommendations of the ClinGen Variant Classification Guidance [17].

#### ALS phenotypes

A three-determinant anatomical classification of ALS motor phenotypes was used which distinguishes between the region of onset (O), the propagation of motor symptoms (P), and the degree of upper (UMN) and/or lower motor neuron (LMN) dysfunction (M), named OPM classification [18]. Phenotypes of onset differentiated the site of first symptoms including the head (“bulbar onset”), arm (“limb onset”), trunk (“thoracic onset”) or leg region (“limb onset”). Phenotypes of propagation differentiated the temporal and spatial pattern in which motor neuron dysfunction spreads from the region of onset to another vertically located body region. Propagation phenotypes included late propagation variants of flail arm and flail leg syndrome. Phenotypes of motor neuron dysfunction distinguished balanced UMN and LMN dysfunction, dominant UMN or pure UMN dysfunction, and dominant or pure LMN dysfunction. The OPM classification system was introduced in April 2025 [18]. Therefore, the phenotype classification according to the OPM was performed retrospectively. Patients with incomplete OPM classification were indicated (Supplementary Document 2). The study focused on motor phenotypes. Thus, cognitive and behavioral phenotypes were not systematically analyzed.

#### ALS functional rating scale (ALSFRS-R)

The ALSFRS-R is a 12-item disease-specific instrument that measures functional impairment in ALS [15].

#### ALS progression rate (ALSPR)

ALSPR was measured by the monthly change of ALSFRS-R scale points and calculated using the following formula: (48 minus ALSFRS-R total score divided by disease duration (months)) [19].

#### Classification of ALSPR

A classification of ALSPR of slower progressing ALS (< 0.5 ALSFRS-R/month), intermediate progressing ALS (≥ 0.5 and ≤ 1.0 ALSFRS-R/month) and faster progressing ALS (> 1.0 ALSFRS-R/month) was applied as previously described [10, 20].

#### Neurofilament light chain in serum (NfL)

The measurement of sNfL concentration was given in pg/mL.

#### NfL in relation to ALSPR (NfL-ALSPR Index)

The NfL-ALSPR ratio serves to normalize the NfL elevation for different ALSPR. Serum NfL was related to ALSPR by dividing the serum NfL concentration (pg/mL) by the value of ALSPR (monthly change of ALSFRS-R scale points), both are strictly positive in the analytic cohort (Eq. 1). To reduce positive skewness and linearise the association, we used the natural logarithm of the ratio (Eq. 2).

Although the raw ratio retains physical units (pg·mL⁻^1^ per ALSFRS-R scale-point·month⁻^1^), the index is log-transformed, which distorts the original units and can invite misinterpretation. For clarity and comparability we therefore report the NFL-ALSPR index as dimensionless.

1 $$NfL(index)=\frac{NfL}{ALSPR}$$

2 $$\mathrm{log}NfL(index)=\mathrm{log}\frac{NfL}{ALSPR}=\text{ log}NfL-\mathrm{log}ALSPR$$

### Statistical methods

All data were analyzed using Prism v10 (GraphPad software). Figures were assembled and formatted in Adobe Illustrator (Adobe Inc.); only layout and typography were adjusted, with no changes to the underlying data. Data are presented as mean ± standard deviation. The applied statistical test is specified in the figure legend. Data comparison with p values of ≤ 0.05 was considered statistically significant. (ns indicates not significant, p > 0.05; * p ≤ 0.05; ** p ≤ 0.01; *** p ≤ 0.001; **** p ≤ 0.0001).

For some patients, not all information was available for the respective categories. The number of patients included in each category is specified accordingly.

## Results

### Frequency of genetic variants

**Fig. 1:**
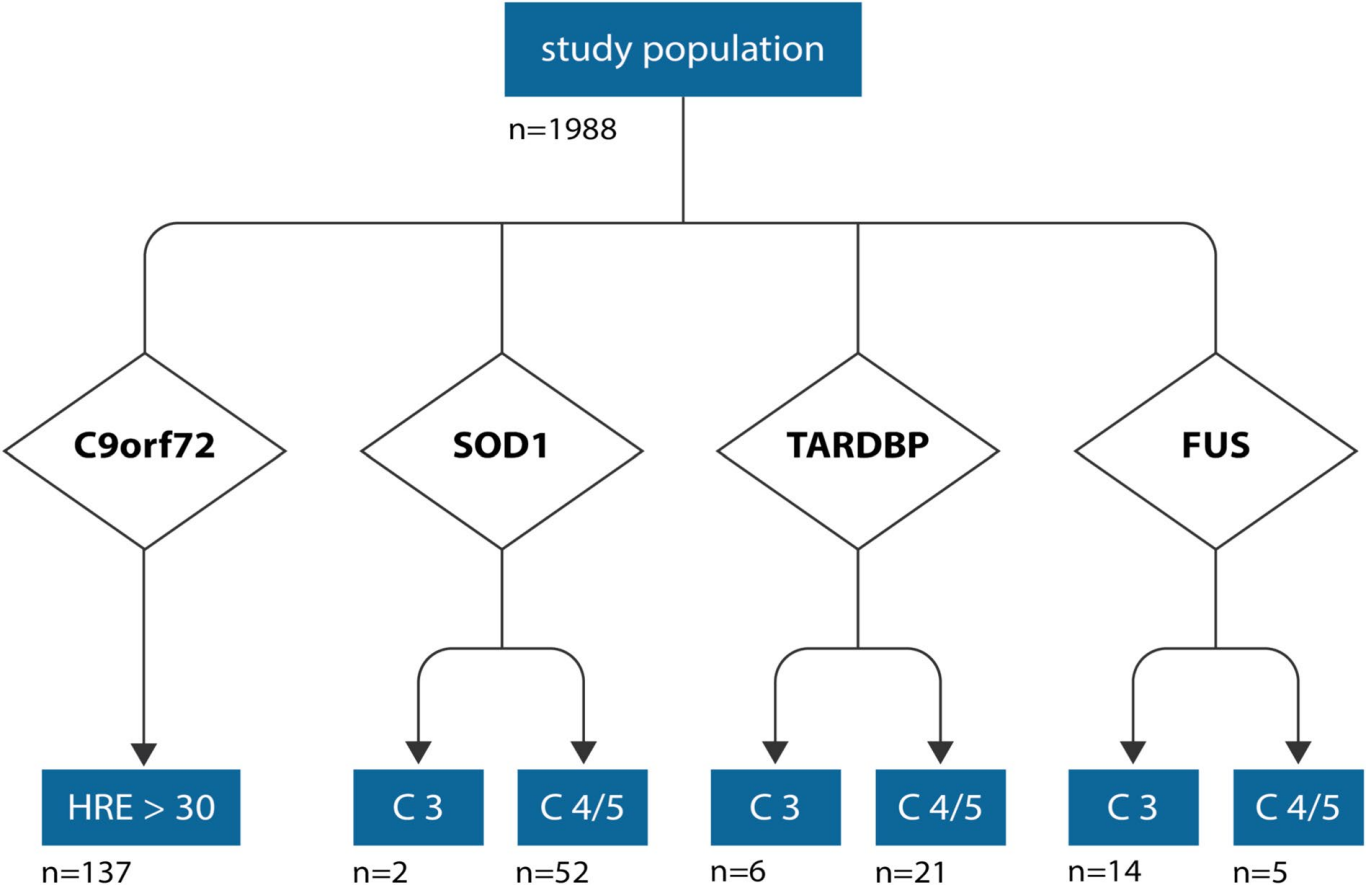
Studied cohort. n = number of ALS patients with genetic variants. C5, pathogenic (class 5) variant; C4, likely pathogenic (class 4) variant; C3, variant of uncertain significance (class 3) according to the guidelines of the American College of Medical Genetics and Genomics (ACMG); HRE: number of hexanucleotide repeat expansion. Only symptomatic carriers had been analyzed further

The frequency of genetic variants is given in Table 1 and Fig. 1, and were in part published elsewhere [14]. Detailed information about the genetic variants can be found in Supplementary Document 1.

**Table 1:** Clinical characteristics, neurofilament and disease progression

	*C9orf72*	*SOD1*	*TARDBP*	*FUS*	*All*
Demographics					
Patients (n)	137	54	27	19	237
Age (years)	59.0 ± 9.2	50.8 ± 11.0	61.9 ± 10.2	57.0 ± 13.9	57.3 ± 10.76
Male	55.5%	44.4%	55.6%	57.9%	53.1%
Female	44.5%	55.6%	44.4%	42.1%	46.9%
Onset					
Patients (n)	122	51	24	17	214
Head region onset (O1)	42.6%	2.0%	4.2%	11.8%	26,2%
Limb onset (O2, O4)	57.4%	96.1%	95.8%	82.4%	72,9%
Trunk onset (O3)	0	2.0%	0	5.9%	0,93%
Propagation					
Early (PE)	76%	70,6%	83,3%	52,9%	73,7%
Late (PL)	11,2%	29,4%	16,7%	47,1%	18,9%
Not specified (PX)	12,8%	0%	0%	0%	7,4%
Degree of upper and lower motor neuron dysfunction					
Balanced UMN and LMN (M0)	74.4%	52.9%	45.8%	23.5%	62,2%
Dominant LMN (M2d)	8.8%	39.2%	29.2%	70.6%	23,0%
Dominant UMN (M1d)	14.4%	7.8%	8.3%	0%	11,01%
Dissociated (M3)	2.4%	0%	16.7%	5.9%	3,7%
Serum neurofilament light chain (sNfL)					
Patients (n)	129	46	25	17	217
NfL (pg/mL)	116.3 ± 72.7	76.1 ± 61.4	43.3 ± 31.8	66.2 ± 32.9	95.43 ± 65.43
ALS progression rate (ALSPR)					
Patients (n)	133	51	23	18	225
ALSPR	0.95 ± 0.84	0.57 ± 0.60	0.53 ± 0.52	0.84 ± 0.85	0.82 ± 0.78
Slower (ALSPR < 0.5)	33.1%	66.7%	69.6%	55.6%	46.2%
Intermediate (ALSPR ≥ 0.5 and ≤ 1.0)	37.6%	11.8%	21.8%	22.2%	28.8%
Faster (ALSPR > 1.0)	29.3%	21.6%	8.7%	22.2%	22.2%
log NfL (index)					
Patients (n)	113	44	21	16	194
log NfL (index)	5.02 ± 0.88	4.94 ± 1.03	4.40 ± 0.7	4.65 ± 0.9	4.90 ± 0.91

Data are presented as mean ± standard deviation. Due to rounding, the sum of values may not always equal 100%

*ALS* amyotrophic lateral sclerosis, *ALSPR* ALS progression rate, *LMN* lower motor neuron, *UMN* upper motor neuron, *sNfL* serum neurofilament light chain, *M0* balanced UMN and LMN dysfunction, *M1d* dominant UMN dysfunction, *M2d* dominant LMN dysfunction, *M3* dissociated motor neuron dysfunction [18]

### Demographic and clinical characteristics

**Fig. 2:**
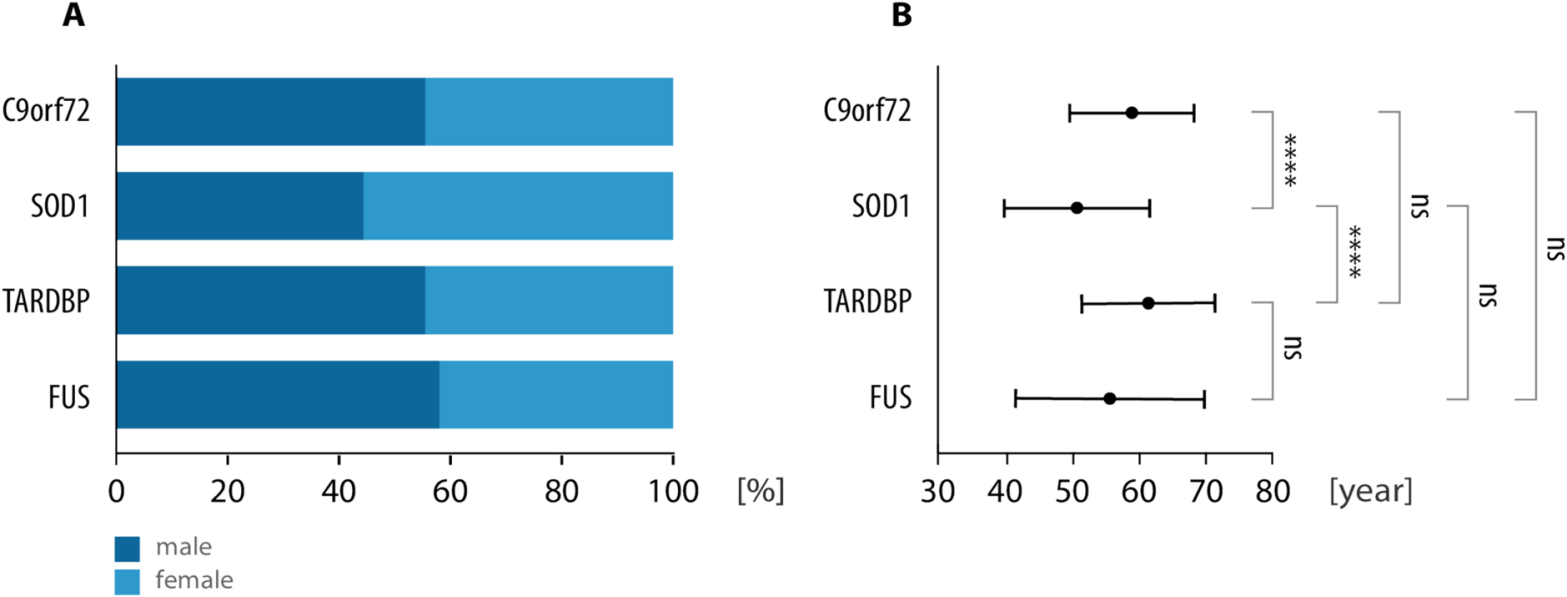
Gender and age distribution across ALS-associated gene mutations. **A** The percentage of male and female patients is displayed for each mutation: C9orf72 (76 male, 55.5%; 61 female, 44.5%), SOD1 (24 male, 44.4%; 30 female, 55.6%), TARDBP (15 male, 55.6%; 12 female, 44.4%), and FUS (11 male, 57.9%; 8 female, 42.1%). Differences in the group and between the groups are not statistically significant (n.s., Chi-Square-Test). **B** Results of the ANOVA test comparing mean ages of patients with mutations C9orf72 (59.0 ± SD 9.2 years, n = 137), SOD1 (50.8 ± SD 11.0 years, n = 54), TARDBP (61.9 ± SD 10.2 years, n = 27), and FUS (57.0 ± SD 13.9 years, n = 19). Data are presented as mean age at disease onset for each subgroup with standard deviations. ns  not significant

Demographic and clinical characteristics of the total cohort are given in Table 1 and were in part published elsewhere [14]. The gender distribution across genetic ALS patients is summarized in Fig. 2. Differences between groups were not statistically significant. Symptom presentation in patients with *SOD1* mutations was significantly earlier than in those with *C9orf72* or *TARDBP* mutations (Table 1). Patients with *FUS* mutations showed a high variability in age at symptom onset ranging from 23 to 72 years. The proportion of familial cases ranged from 21.1% (*FUS*, n = 19) to 40.7% (*SOD1*, n = 54), emphasizing that a positive family history is less likely than apparently sporadic ALS across all studied forms of genetic ALS. Also, *C9orf72* ALS, the most frequent genetic ALS, was predominantly associated with a negative family history (72.3%, n = 137).

### ALS phenotypes

**Fig. 3:**
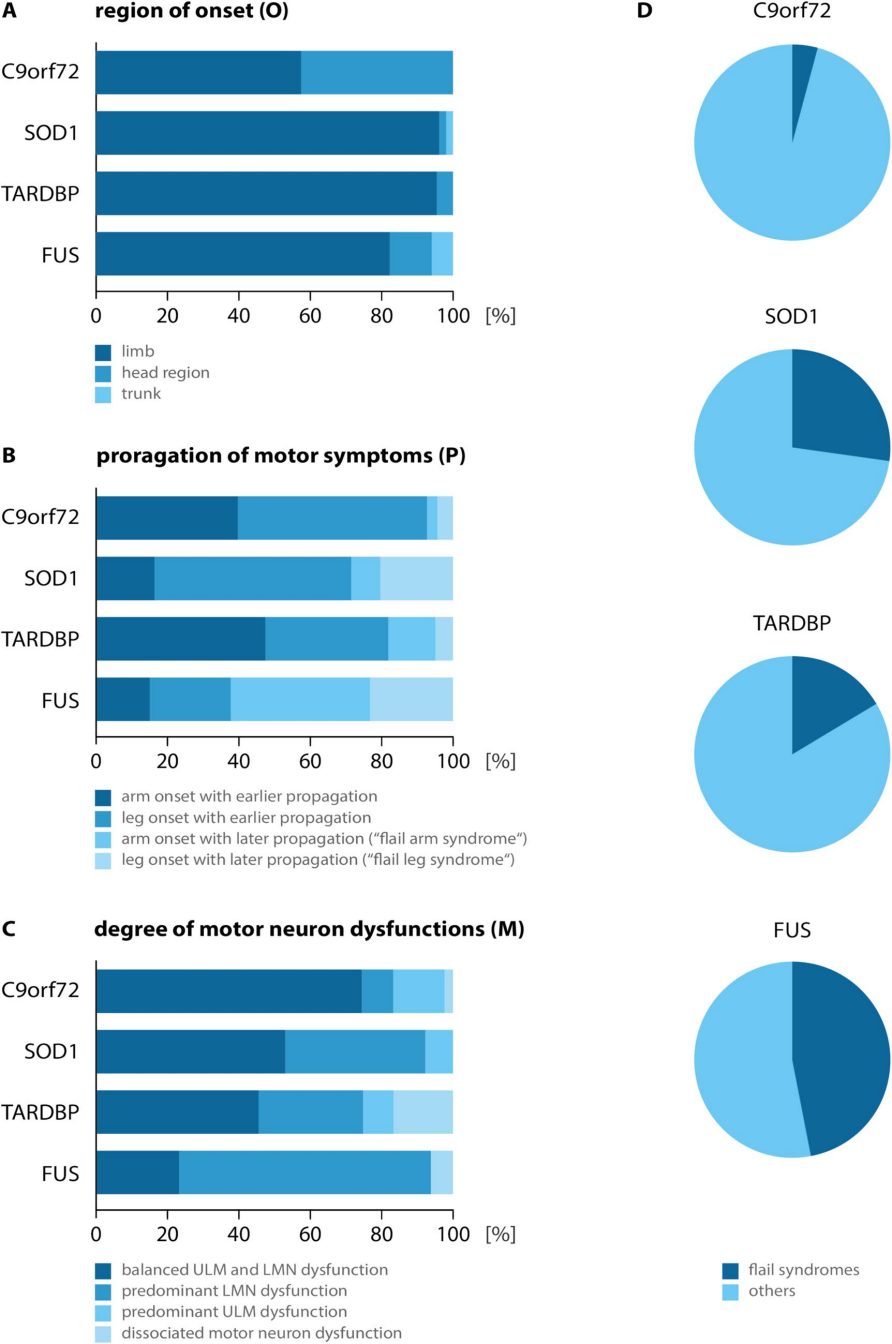
Distribution of clinical phenotypes and motor neuron involvement. **A** Proportional distribution of region onset localization (limb, head region, trunk) across the genetic subgroups *C9orf72* (57.4% limb, 42.6% head region, 0% trunk, n = 122), *SOD1* (96.1% limb, 2.0% head region, 2.0% trunk, n = 51), *TARDBP* (95.8% limb, 4.2% head region, 0% trunk, n = 24), and *FUS* (82.4% limb, 11.8% head region, 5.9% trunk, n = 17). **B** Distribution of propagation of motor symptoms for arm onset and earlier propagation, leg onset and earlier propagation, arm onset with later propagation (“flail arm syndrome”) and leg onset with later propagation (“flail leg syndrome”) are shown for *C9orf72* (39.7%, 53.0%, 2.9%, 4.4%, n = 68), *SOD1* (16.3%, 55.1%, 8.2%, 20.4%, n = 49), *TARDBP *(47.8%, 34.8%, 13.0%, 4.3%, n = 23), and *FUS* (15.4%, 23.1%, 38.4%, 23.1%, n = 13). **C** Distribution of motor neuron dysfunction of patients with balanced UMN and LMN dysfunction, predominant LMN dysfunction, predominant UMN dysfunction and dissociated motor neuron dysfunction is shown for *C9orf72* (74.4%, 8.8%, 14.4%, 2.4%, n = 122), *SOD1* (52.9%, 39.2%, 7.8%, 0%, n = 51), *TARDBP* (45.8%, 29.2%, 8.3%, 16.7%, n = 24) and *FUS* (23.5%, 70.6%, 0%, 5.9%, n = 17). **D** Proportion of flail variants compared to all phenotypes including non-limb are shown for *C9orf72* (4.1%), *SOD1* (27.5%), *TARDBP* (16.7%) and *FUS* (47.1%)

Phenotyping was performed according to the OPM classification (Fig. 3, Supplementary Document 2) [18]. In *C9orf72*-ALS, limb onset was found in 57.4% of patients, closely followed by onset in the head region (42.6%). *C9orf72*-ALS patients revealed the highest prevalence of head onset (“bulbar onset”) across all studied genetic ALS. In comparison, patients with *FUS*-ALS predominantly exhibited limb onset phenotypes (82.4%). Patients with *SOD1* and *TARDBP* mutations demonstrated a striking predominance of limb onset (96.1 and 95.8%, respectively). Leg onset dominated (75.5%) *SOD1*-ALS, while arm onset was more frequent (60.9%) in *TARDBP*-ALS. When classifying for propagation, only 4.1% of all *C9orf72*-ALS showed late limb propagation of motor neuron dysfunction (“flail arm” or “flail leg” syndromes), making this a rare phenotype (Fig. 3D). Remarkably, late propagation phenotypes (flail limb variants) accounted for 47.1% of *FUS*-ALS, representing the highest proportion among genetic ALS. Phenotyping for motor neuron dysfunction, *C9orf72*-ALS showed a balanced UMN and LMN involvement in most (74.4%) patients whereas the LMN predominant phenotype was uncommon (8.8%). In *SOD1-* and *TARDBP*-ALS, a balanced motor involvement was only found in 52.9 and 45.8%, respectively, followed by a high frequency of the predominant LMN phenotype (39.2 and 29.2%, respectively). Moreover, *FUS*-ALS displayed a strikingly high proportion of predominant LMN dysfunction (70.6%).

### ALS progression rate (ALSPR)

**Fig. 4:**
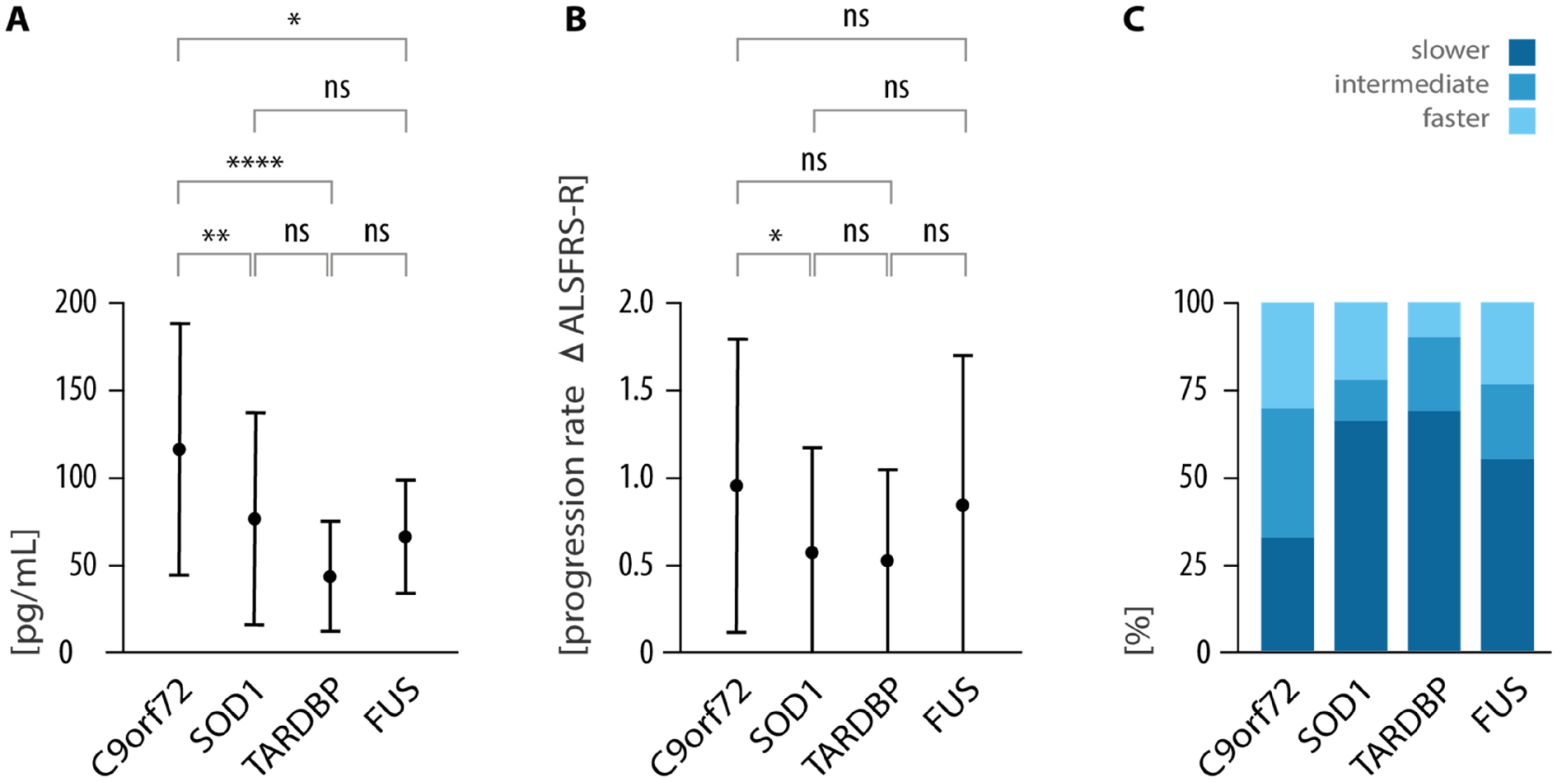
Disease activity displayed by sNfL and progression rate. **A** Shown are sNFL levels as mean values and standard deviation: *C9orf72* group (116.3 ± SD 72.7 pg/mL, n = 129); *SOD1* (76.1 ± SD 61.4 pg/mL, n = 46); *TARDBP* (43.3 ± SD 31.8 pg/mL, n = 25); and *FUS* (66.2 ± SD 32.9 pg/mL, n = 17). **B** Mean progression rates of the ALS-FR-R. The mean values are as follows: *C9orf72* (0.95 ± 0.84, n = 133); *SOD1* (0.57 ± 0.6, n = 51); *TARDBP* (0.53 ± 0.52, n = 23); and *FUS* (0.84 ± 0.85, n = 18). **C** Distribution of progression groups among *C9orf72* (33.1% slower, 37.6% intermediate, 29.3% faster), *SOD1* (66.7% slower, 11.8% intermediate, 21.6% faster), *TARDBP* (69.6% slower, 21.8% intermediate, 8.7% faster), and *FUS* (55.6% slower, 22.2% intermediate, 22.2% faster)

Patients with *C9orf72* ALS showed the fastest progression among genetic ALS (ALSPR mean 0.95, SD 0.84) (Fig. 4B). ALSPR revealed a broad spectrum of slower (33.1%), intermediate (37.6%), and faster ALS progression (29.3%) (Fig. 4C). In contrast, patients with *SOD1*-ALS demonstrated a slower progression (ALSPR mean 0.57, SD 0.60), with 66.7% of patients meeting the criteria of slower ALS progression. Besides the group of slower progressing *SOD1*-ALS, 21.6% of patients showed faster and 11.8% intermediate ALS progression. *TARDBP* mutation exhibited the slowest ALS progression (ALSPR mean 0.53, SD 0.52) with 69.6% of patients in the slower ALS progression category, followed by 21.8% intermediate, and 8.7% faster progressing ALS. *FUS*-ALS patients showed a mean ALSPR of 0.84 (SD 0.85). However, most *FUS*-ALS patients experienced slower progression (55.6%), with intermediate and faster courses equally represented (22.2%).

### Neurofilament light chain in serum (NfL)

Patients with *C9orf72*-ALS had the highest sNfL levels (mean 116.3, SD 72.7 pg/mL) (Fig. 4A). In contrast, *SOD1*-ALS showed a mean concentration of 76.1 pg/mL, reflecting the broad spectrum of disease progression. *TARDBP* mutation carriers exhibited the lowest sNfL levels (mean 43.3 pg/mL), whereas in *FUS*-ALS a moderate sNfL elevation (mean 66.2 pg/mL) was found.

### NfL-ALSPR index

The NfL-ALSPR index for *C9orf72* ALS was the highest among all investigated genetic ALS (5.02 ± 0.88), followed by *SOD1* ALS (4.94 ± 1.03), *FUS* (4.9 ± 0.91) and *TARDBP* (4.4 ± 0.7) (Table 1). A detailed subanalysis of the logNfL(index) across individual phenotypic variables, pooling all mutation carriers into a single cohort, is provided in Supplementary Document 3.

## Discussion

This multicenter study emphasized distinct clinical phenotypes, ALS progressions and the NfL profiles in genetic ALS. This study included ALS patients regardless of family history. This unbiased approach enables a more comprehensive understanding of genetic ALS phenotypes as previous studies have predominantly focused on familial ALS cases. Notably, genetic ALS was predominantly identified in patients with apparently sporadic disease, consistent with earlier reports [14]. This study included class 3 variants of uncertain significance (VUS) in *SOD1, TARDBP* and *FUS*. The rationale was the current inability to definitively assess the pathogenicity of these variants. This inclusion is of particular relevance in the context of emerging targeted therapies [5, 21]. Given their therapeutic potential, even uncertain variants may merit consideration for early treatment inclusion [21]. Historically, definitive pathogenic classification of *SOD1* and *FUS* mutations was often only possible postmortem, through the identification of the respective inclusion bodies. With the advent of disease-modifying treatments, therapeutic response—such as a decline in NfL levels—may serve as a surrogate marker for pathogenicity [22].

Overall, genetic ALS is associated with a younger age of onset compared to non-genetic ALS, while sex distribution does not differ significantly. In particular, patients with *SOD1* mutations exhibited markedly earlier symptom onset, with a mean age approximately 10 years younger than that of the total cohort, consistent with previous epidemiologic reports on ALS demographics [5, 23]. In contrast, patients with *C9orf72* or *TARDBP* mutations showed onset ages comparable to those observed in sporadic ALS, in line with recent studies [14]. Notably, *FUS*-associated ALS demonstrated high variability in age at symptom onset, ranging from 23 to 72 years, encompassing both juvenile and late-onset cases [21]. This study underscores that *FUS* ALS is not confined to early-onset forms but spans a broad age range at disease onset.

Genetic ALS displays distinct phenotypic characteristics, consistent with previous reports [2]. In this study, phenotypes were characterized in greater detail based on three anatomical determinants: region of onset, propagation pattern of motor dysfunction, and the relative degree of upper and/or lower motor neuron involvement [18]. Among all genetic ALS subtypes, *C9orf72*-associated ALS showed the highest prevalence of bulbar onset ("head onset", O1), closely resembling the distribution observed in sporadic ALS. This pattern is consistent with prior studies describing *C9orf72* ALS as clinically proximate to non-genetic ALS [24–26]. In contrast, patients with *FUS* mutations predominantly presented with limb onset phenotypes (81%), as previously observed [27, 28]. *SOD1*- and *TARDBP*-associated ALS showed an even stronger predominance of limb onset (96 and 95%, respectively). Leg onset dominated in *SOD1* ALS, whereas *TARDBP* ALS more frequently began in the arms, aligning with earlier phenotype descriptions in these genotypes [29–31].

The pattern of propagation is an important phenotypic determinant with prognostic relevance in ALS. In *C9orf72*-associated ALS, motor neuron dysfunction most frequently followed an early vertical propagation pattern. Flail limb phenotypes—representing late propagation patterns—were rare, occurring in only 4.1% of cases. In contrast, flail limb variants were observed in 47.1% of FUS- ALS, a proportion substantially higher than in the total ALS cohort [11]. Notably, this propagation pattern has not been systematically investigated in prior *FUS*-ALS studies and requires validation in independent cohorts and populations.

Our findings demonstrate genotype-specific predominance of UMN and LMN involvement in genetic ALS. *C9orf72*-ALS was characterized by a predominantly balanced UMN and LMN involvement (M0) in the majority of patients (74.4%), while LMN-dominant phenotypes (M2d, M2p) were uncommon. In contrast, *SOD1-*ALS showed a LMN-predominance, in alignment with previous reports [29, 32]. Also, *FUS*-ALS demonstrated a striking predominance of LMN-dominant phenotypes (70.6%), consistent with earlier observations in smaller series [27, 28]. Interestingly, *TARDBP*-ALS exhibited a remarkably high frequency of the M3 phenotype, a pattern not observed in other genetic ALS [30, 33].

Genetic ALS subtypes differ in their overall progression dynamics as measured by the ALSPR. *C9orf72*-ALS exhibited the most rapid disease progression (mean ALSPR: 0.95), followed by *FUS*, *SOD1*, and *TARDBP*-associated ALS. However, within each genotype, a wide range of individual ALSPR were observed. Classification based on ALSPR helps to generate a more differentiated view of disease trajectories [34]. Thus, although the overall group of *C9orf72*-ALS is associated with faster ALS progression, one third of patients in this group exhibited slower disease progression. Similarly, a more nuanced approach is needed in *SOD1*-ALS, which is generally linked to a more favorable prognosis (mean ALSPR: 0.57). However, a substantial subset of patients showed faster (21.6%) or intermediate (11.8%) disease progression. A similar observation was found in *TARDBP*-ALS, that demonstrated an overall slower ALS progression (mean ALSPR 0.53). At the same time, a relevant group of patients revealed faster (8.7%) or intermediate (21.8%) ALSPR. Prior reports describe *FUS*-ALS as early-onset and highly aggressive [35]. However, this study reveals substantial heterogeneity in age at onset and disease progression, suggesting a broader clinical spectrum than previously assumed. FUS-associated ALS is the most heterogeneous form of genetic ALS, with an overall ALSPR indicating more aggressive disease progression (mean ALSPR 0.84), referring to patients with intermediate (22.2%) and faster (22.2%) progression. Conversely, 55.6% of patients experienced slower progression. In conclusion, distinguishing different trajectories in any form of genetic ALS is essential for diagnosis, counseling, and designing clinical trials for diseases caused by ALS-associated genes.

This is the first systematic study specifically addressing NfL concentrations across different forms of genetic ALS. Patients with *C9orf72*-ALS demonstrated the highest NfL levels (mean 116.3 pg/mL). This finding corresponds with high ALSPR and high proportion of bulbar onset ALS, both known contributors of NfL elevation in ALS [10, 11]. Patients with *SOD1-ALS* were the youngest in the studied cohort and exhibited a relatively slow disease progression. Both factors—young age and slow progression—are typically associated with lower NfL levels. However, in this study, *SOD1*-ALS patients displayed moderate rather than particularly low concentrations (mean 76.1 pg/mL). *TARDBP* mutation carriers exhibited the lowest NfL levels (mean 43.4 pg/mL). This finding corresponds with predominant limb-onset phenotypes and low ALSPR. In the group of *FUS*-ALS, a moderate NfL elevation (mean 66.2, SD 32.9 pg/mL) was found reflecting the substantial heterogeneity in disease progression.

The systematic phenotyping applied in this study enabled a detailed subgroup analysis of motor phenotypes with respect to NfL levels. The highest NfL concentrations were observed in patients with head region onset (O1), which was also associated with the highest ALS progression rate, consistent with the typically more aggressive disease course in bulbar-onset ALS. As expected, early propagation variants demonstrated higher NfL levels, reflecting the faster rate of disease progression characteristic of these phenotypes. Regarding motor neuron involvement, patients with balanced upper and lower motor neuron dysfunction (M0) exhibited the highest NfL concentrations, likely due to the cumulative neuroaxonal degeneration affecting both motor neuron systems.

Our data revealed a systematic mismatch between sNfL und ALSPR, especially in *SOD1* carriers: despite a slow functional decline (low monthly progression rate), these patients showed disproportionately high sNfL. To quantify this disproportionate sNfL elevation, the NFL-ALSPR index was derived, defined as the log-transformed ratio of sNfL and ALSPR. However, a simple ratio is unsatisfactory because both constituents are right-skewed, therefore their raw ratio tends to be even more skewed. Log-transformation stabilizes the distribution. The ratio allows comparison across mutation groups with different tempos of decline. Uncertainty increases at very high ALSPR where the sNfL correlation widens, so single outliers must be interpreted cautiously. Of note, the index is undefined for ALSPR = 0. However, this issue does not arise in our dataset, as only symptomatic patients were included and ALSPR was therefore never zero. In conclusion, the NFL-ALSPR index condenses two correlated biomarkers into a single value, can be calculated without specialized software and—if required—other covariates could additionally be incorporated for formally adjusted inference. Although individual extremes warrant cautious interpretation, the model can nevertheless be used as a practical and clinically intuitive tool for both research and routine assessment of genotype-specific sNfL deviations.

The NfL-ALSPR index refers to previous reports in which distinct ALS motor phenotypes were found to have contributed independently to NfL elevation [11]. Similarly, it is conceivable that distinct genotypes have a different impact on NfL elevation. *C9orf72*-associated ALS showed the highest logNfL(index) (5.02 ± 0.88), suggesting that *C9orf72*-related disease, which is phenotypically close to sporadic ALS and typically associated with faster progression, shows the strongest proportional NfL elevation relative to functional decline. Remarkably, the logNfL(index) for *SOD1*-ALS was nearly comparable (4.94 ± 1.03). *C9orf72*-associated ALS showed the highest logNfL(index) (5.02 ± 0.88), suggesting that *C9orf72*-related disease, which is phenotypically close to sporadic ALS, shows the strongest proportional NfL elevation relative to functional decline. Remarkably, the logNfL(index) for *SOD1*-ALS was high and close to the values of *C9orf72*-associated ALS (4.94 ± 1.03). The reasons for the comparably strong NfL elevation in *SOD1*-ALS remain unknown. A comparison between the arm (O2) and leg (O4) onset groups revealed no differences in logNfL(index), rendering pathoanatomical characteristics of SOD1-ALS an unlikely explanation for this observation. Therefore, genetically determined disease mechanisms may underlie the elevated logNfL(index) observed in SOD1-ALS. In contrast, the logNfL(index) was rather low in *TARDBP*-ALS (4.40 ± 0.7) and *FUS* (4.65 ± 0.9). Overall, these different NfL/ALSPR ratio results suggest that unknown pathogenetic factors related to the genetic etiology may contribute independently to NfL elevation.

Several limitations of this study must be considered. This investigation included class 3 variants of uncertain significance (VUS). Given the therapeutic relevance of these genes, especially in the context of emerging ASO-based treatments, their inclusion is justified. However, a continuous re-evaluation of class 3 variants, particularly in *SOD1* and *FUS*, is expected, and may prompt adjustments of the analyzed dataset. The subgroups of *TARDBP-* and *FUS-ALS*, although larger than in most previous reports, remains relatively small, limiting the power to detect nuanced differences in phenotypes, ALSPR and NfL levels. Furthermore, longitudinal analyses of genetic ALS cohorts will be essential to confirm our findings. Furthermore, *C9orf72* is the predominant mutation in European ALS cohorts. Therefore, the epidemiological data, collected exclusively in Germany, may not be generalizable to other populations. Additionally, while we focused on the most common ALS-causing genes, other rare variants and polygenic factors may contribute to ALS susceptibility and progression, warranting broader genomic exploration in future studies. Finally, since the analysis of the logNfL(index) was restricted to mutation carriers, comparisons across the broader ALS population, particularly with sporadic ALS, were not possible. Consequently, observed differences in the index across the analyzed variables may be influenced by the distribution of these genotypes. Thus, the current data allow conclusions only within the context of genetic ALS. Future analyses in larger cohorts, including sporadic ALS, are warranted to validate the logNfL(index) as an integrative biomarker readout. Such efforts are already planned within the framework of the ongoing multicenter study on NfL and ALS phenotypes [11].

In conclusion, this study underscores the phenotypic and biomarker heterogeneity across and within distinct forms of genetic ALS. Our results advocate for further investigate NfL to better understand the impact of specific genotypes on NfL elevation. By integrating detailed phenotyping, biomarker data and a systematic screening approach, these findings pave the way for a more personalized approach in genetic counselling, the refinement of prediction models for interventional trials, and the deepening of our pathogenetic understanding in genetic ALS.

## Supplementary Information

Below is the link to the electronic supplementary material.

Supplementary file1 (DOCX 30 KB)

Supplementary file2 (DOCX 63 KB)

Supplementary file3 (DOCX 17 KB)

## Data Availability

The data that support the findings of this study are available from the corresponding author upon reasonable request.

## References

[1] Brown RH, Al-Chalabi A (2017) Amyotrophic lateral sclerosis. N Engl J Med 377:162–172. 10.1056/NEJMra1603471 28700839 10.1056/NEJMra1603471

[2] Goutman SA, Hardiman O, Al-Chalabi A et al (2022) Emerging insights into the complex genetics and pathophysiology of amyotrophic lateral sclerosis. Lancet Neurol 21:465–479. 10.1016/S1474-4422(21)00414-2 35334234 10.1016/S1474-4422(21)00414-2 PMC9513754

[3] Ruf WP, Boros M, Freischmidt A et al (2023) Spectrum and frequency of genetic variants in sporadic amyotrophic lateral sclerosis. Brain Commun 5:fcad152. 10.1093/braincomms/fcad152 37223130 10.1093/braincomms/fcad152 PMC10202555

[4] Miller TM, Cudkowicz ME, Genge A et al (2022) Trial of antisense oligonucleotide tofersen for SOD1 ALS. N Engl J Med 387:1099–1110. 10.1056/NEJMoa2204705 36129998 10.1056/NEJMoa2204705

[5] Meyer T, Schumann P, Weydt P et al (2023) Neurofilament light-chain response during therapy with antisense oligonucleotide tofersen in SOD1-related ALS: treatment experience in clinical practice. Muscle Nerve 67:515–521. 10.1002/mus.27818 36928619 10.1002/mus.27818

[6] Hamad AA, Alkhawaldeh IM, Nashwan AJ et al (2025) Tofersen for SOD1 amyotrophic lateral sclerosis: a systematic review and meta-analysis. Neurol Sci 46:1977–1985. 10.1007/s10072-025-07994-2 39820998 10.1007/s10072-025-07994-2 PMC12003547

[7] Jiang Q, Lin J, Wei Q et al (2024) Amyotrophic lateral sclerosis patients with various gene mutations show diverse motor phenotypes and survival in China. J Med Genet 61:839–846. 10.1136/jmg-2024-109909 38886047 10.1136/jmg-2024-109909

[8] McFarlane R, Opie-Martin S, Caravaca Puchades A et al (2025) Clinical trajectories of genetic variants in ALS: a European observational study within PRECISION-ALS. Amyotroph Lateral Scler Frontotemporal Degener 26:41–49. 10.1080/21678421.2025.2450805 40326912 10.1080/21678421.2025.2450805

[9] Thouvenot E, Demattei C, Lehmann S et al (2020) Serum neurofilament light chain at time of diagnosis is an independent prognostic factor of survival in amyotrophic lateral sclerosis. Eur J Neurol 27:251–257. 10.1111/ene.14063 31437330 10.1111/ene.14063

[10] Meyer T, Salkic E, Grehl T et al (2023) Performance of serum neurofilament light chain in a wide spectrum of clinical courses of amyotrophic lateral sclerosis-a cross-sectional multicenter study. Eur J Neurol 30:1600–1610. 10.1111/ene.15773 36899448 10.1111/ene.15773

[11] Meyer T, Dreger M, Grehl T et al (2024) Serum neurofilament light chain in distinct phenotypes of amyotrophic lateral sclerosis: a longitudinal, multicenter study. Eur J Neurol 31:e16379. 10.1111/ene.16379 38859579 10.1111/ene.16379 PMC11295170

[12] Shefner JM, Al-Chalabi A, Baker MR et al (2020) A proposal for new diagnostic criteria for ALS. Clin Neurophysiol 131:1975–1978. 10.1016/j.clinph.2020.04.005 32387049 10.1016/j.clinph.2020.04.005

[13] Meyer T, Spittel S, Grehl T et al (2023) Remote digital assessment of amyotrophic lateral sclerosis functional rating scale—a multicenter observational study. Amyotroph Lateral Scler Frontotemporal Degener 24:175–184. 10.1080/21678421.2022.2104649 35912984 10.1080/21678421.2022.2104649

[14] Meyer T, Schumann P, Grehl T et al (2025) SOD1 gene screening in ALS—frequency of mutations, patients’ attitudes to genetic information and transition to tofersen treatment in a multi-center program. Amyotroph Lateral Scler Frontotemporal Degener 26:162–171. 10.1080/21678421.2024.2401131 39268612 10.1080/21678421.2024.2401131

[15] Cedarbaum JM, Stambler N, Malta E et al (1999) The ALSFRS-R: a revised ALS functional rating scale that incorporates assessments of respiratory function. J Neurol Sci 169:13–21. 10.1016/s0022-510x(99)00210-5. (**BDNF ALS Study Group (Phase III)**) 10540002 10.1016/s0022-510x(99)00210-5

[16] Richards S, Aziz N, Bale S et al (2015) Standards and guidelines for the interpretation of sequence variants: a joint consensus recommendation of the American college of medical genetics and genomics and the association for molecular pathology. Genet Med 17:405–424. 10.1038/gim.2015.30 25741868 10.1038/gim.2015.30 PMC4544753

[17] Harrison SM, Riggs ER, Maglott DR et al (2016) Using ClinVar as a resource to support variant interpretation. Curr Protoc Hum Genet 89 8–16.1–8.16–23. 10.1002/0471142905.hg0816s89 10.1002/0471142905.hg0816s89 PMC4832236 27037489

[18] Meyer T, Boentert M, Großkreutz J et al (2025) Motor phenotypes of amyotrophic lateral sclerosis—a three-determinant anatomical classification based on the region of onset, propagation of motor symptoms, and the degree of upper and lower motor neuron dysfunction. Neurol Res Pract 7:27. 10.1186/s42466-025-00389-w 40289140 10.1186/s42466-025-00389-w PMC12036282

[19] Kimura F, Fujimura C, Ishida S et al (2006) Progression rate of ALSFRS-R at time of diagnosis predicts survival time in ALS. Neurology 66:265–267. 10.1212/01.wnl.0000194316.91908.8a 16434671 10.1212/01.wnl.0000194316.91908.8a

[20] Benatar M, Zhang L, Wang L et al (2020) Validation of serum neurofilaments as prognostic and potential pharmacodynamic biomarkers for ALS. Neurology 95:e59–e69. 10.1212/WNL.0000000000009559 32385188 10.1212/WNL.0000000000009559 PMC7371380

[21] Shneider NA, Harms MB, Korobeynikov VA et al (2025) Antisense oligonucleotide jacifusen for FUS-ALS: an investigator-initiated, multicentre, open-label case series. Lancet 405:2075–2086. 10.1016/S0140-6736(25)00513-6 40414239 10.1016/S0140-6736(25)00513-6 PMC12407188

[22] Weishaupt JH, Körtvélyessy P, Schumann P et al (2024) Tofersen decreases neurofilament levels supporting the pathogenesis of the *SOD1* p.D91A variant in amyotrophic lateral sclerosis patients. Commun Med 4:150. 10.1038/s43856-024-00573-0 39054363 10.1038/s43856-024-00573-0 PMC11272917

[23] Wiesenfarth M, Forouhideh-Wiesenfarth Y, Elmas Z et al (2024) Clinical characterization of common pathogenic variants of SOD1-ALS in Germany. J Neurol 271:6667–6679. 10.1007/s00415-024-12564-1 39141064 10.1007/s00415-024-12564-1 PMC11446975

[24] Colombo E, Poletti B, Maranzano A et al (2023) Motor, cognitive and behavioural profiles of C9orf72 expansion-related amyotrophic lateral sclerosis. J Neurol 270:898–908. 10.1007/s00415-022-11433-z 36308529 10.1007/s00415-022-11433-z PMC9886586

[25] Chiò A, Borghero G, Restagno G et al (2012) Clinical characteristics of patients with familial amyotrophic lateral sclerosis carrying the pathogenic GGGGCC hexanucleotide repeat expansion of C9ORF72. Brain 135:784–793. 10.1093/brain/awr366 22366794 10.1093/brain/awr366 PMC3286333

[26] Wiesenfarth M, Günther K, Müller K et al (2023) Clinical and genetic features of amyotrophic lateral sclerosis patients with C9orf72 mutations. Brain Commun 5:fcad087. 10.1093/braincomms/fcad087 37006326 10.1093/braincomms/fcad087 PMC10065188

[27] Grassano M, Brodini G, De Marco G et al (2022) Phenotype analysis of fused in sarcoma mutations in amyotrophic lateral sclerosis. Neurol Genet 8:e200011. 10.1212/NXG.0000000000200011 36105853 10.1212/NXG.0000000000200011 PMC9469212

[28] Naumann M, Peikert K, Günther R et al (2019) Phenotypes and malignancy risk of different FUS mutations in genetic amyotrophic lateral sclerosis. Ann Clin Transl Neurol 6:2384–2394. 10.1002/acn3.50930 31682085 10.1002/acn3.50930 PMC6917314

[29] Benatar M, Robertson J, Andersen PM (2025) Amyotrophic lateral sclerosis caused by SOD1 variants: from genetic discovery to disease prevention. Lancet Neurol 24:77–86. 10.1016/S1474-4422(24)00479-4 39706636 10.1016/S1474-4422(24)00479-4

[30] Corcia P, Valdmanis P, Millecamps S et al (2012) Phenotype and genotype analysis in amyotrophic lateral sclerosis with TARDBP gene mutations. Neurology 78:1519–1526. 10.1212/WNL.0b013e3182553c88 22539580 10.1212/WNL.0b013e3182553c88

[31] Domi T, Schito P, Sferruzza G et al (2024) Unveiling the SOD1-mediated ALS phenotype: insights from a comprehensive meta-analysis. J Neurol 271:1342–1354. 10.1007/s00415-023-12074-6 37930481 10.1007/s00415-023-12074-6

[32] Berdyński M, Miszta P, Safranow K et al (2022) SOD1 mutations associated with amyotrophic lateral sclerosis analysis of variant severity. Sci Rep 12:103. 10.1038/s41598-021-03891-8 34996976 10.1038/s41598-021-03891-8 PMC8742055

[33] Lombardi M, Corrado L, Piola B et al (2023) Variability in clinical phenotype in TARDBP mutations: amyotrophic lateral sclerosis case description and literature review. Genes. 10.3390/genes14112039 38002982 10.3390/genes14112039 PMC10671725

[34] Alves I, Gromicho M, Oliveira Santos M et al (2025) Assessing disease progression in ALS: prognostic subgroups and outliers. Amyotroph Lateral Scler Frontotemporal Degener 26:58–63. 10.1080/21678421.2024.2407412 39340290 10.1080/21678421.2024.2407412

[35] Yan J, Deng H-X, Siddique N et al (2010) Frameshift and novel mutations in FUS in familial amyotrophic lateral sclerosis and ALS/dementia. Neurology 75:807–814. 10.1212/WNL.0b013e3181f07e0c 20668259 10.1212/WNL.0b013e3181f07e0c PMC2938970

