# Supplementary material for: Motor phenotypes and neurofilament light chain in genetic amyotrophic lateral sclerosis—results from a multicenter screening program
Source: J Neurol. 2025 Dec 12;273(1):22. doi: 10.1007/s00415-025-13555-6 (PMC12700978; doi:10.1007/s00415-025-13555-6)
Supplement: Supplementary file 1 — Supplementary file1 (DOCX 30 KB) [file 415_2025_13555_MOESM1_ESM.docx]

| *Patient ID* | *overall comment* | *ACMG classification* | *ACMG criteria* |
| --- | --- | --- | --- |
| SOD#1 | heterozygous for: g.33036152A>G, NM_000454.5(SOD1), c.122A>G, p.(Glu41Gly) | 4 | PS4_str, PM2_sup, PP3_sup |
| SOD#2 | heterozygous for: g.33040802G>T,c.376G>T,p.(Asp126Tyr). | 4 | PS4_sup, PM2_sup, PP3_mod, PM5_mod |
| SOD#3 | heterozygous for: g.33040861G>C, NM_000454.5(SOD1):c.435G>C, p.(Leu145Phe) | 5 | PS4_str, PP1_sup, PS3_sup, PS1_str, PM2_sup, PP3_mod |
| SOD#4 | heterozygous for g.33039677C>G, NM_000454.5(SOD1):c.346C>G, p.(Arg116Gly) | 5 | PS4_str, PM1_mod, PM5_mod,PM2_sup, PP3_mod |
| SOD#5 | heterozygous for: g.33039603A>C, NM_000454.5(SOD1):c.272A>C, p.(Asp91Ala) (D91A) | 4 | PM3_str, PM1_mod, PS3_sup, PM5_sup |
| SOD#6 | homozygous for g.33039603A>C, NM_000454.5(SOD1):c.272A>C, p.(Asp91Ala) | 4 | PM3_str, PM1_mod, PS3_sup, PM5_sup |
| SOD#7 | homozygot for: g.33039603A>C, NM_000454.5(SOD1):c.272A>C, p.(Asp91Ala) | 4 | PM3_str, PM1_mod, PS3_sup, PM5_sup |
| SOD#8 | homozygot for: g.33039603A>C, NM_000454.5(SOD1):c.272A>C, p.(Asp91Ala) | 4 | PM3_str, PM1_mod, PS3_sup, PM5_sup |
| SOD#9 | heterozygot for NM_000454.5(SOD1):c.346C>G/p.(Arg116Gly) | 5 | PS4_str, PM1_mod, PM5_mod,PM2_sup, PP3_mod |
| SOD#10 | heterozygous for: NM_000454.5(SOD1):c.346C>G/p.(Arg116Gly) | 5 | PS4_str, PM1_mod, PM5_mod,PM2_sup, PP3_mod |
| SOD#11 | heterozygous for: g.33039603A>C, NM_000454.5(SOD1):c.272A>C, p.(Asp91Ala) | 4 | PM3_str, PM1_mod, PS3_sup, PM5_sup |
| SOD#12 | heterozygous for NM_000454.5(SOD1):c.125G>A/p.(Gly42Asp) | 5 | PS4_str, PS3_sup, PM2_sup, PM5_mod, PP3_mod |
| SOD#13 | homozygot for g.33039603A>C, NM_000454.5(SOD1):c.272A>C, p.(Asp91Ala | 4 | PM3_str, PM1_mod, PS3_sup, PM5_sup |
| SOD#14 | heterozygous for: NM_000454.5(SOD1):c.346C>G/p.(Arg116Gly) | 5 | PS4_str, PM1_mod, PM5_mod,PM2_sup, PP3_mod |
| SOD#15 | heterozygous for: NM_000454.5(SOD1):c.341T>C/p.(Ile114Thr) | 4 | PS4_mod, PM1_mod, PS3_sup, PP3_mod |
| SOD#16 | heterozygous for: NM_000454.5(SOD1):c.272A>C/ p.(Asp91Ala) | 4 | PM3_str, PM1_mod, PS3_sup, PM5_sup |
| SOD#17 | heterozygous for: NM_000454.5(SOD1):c.346C>G/p.(Arg116Gly) | 5 | PS4_str, PM1_mod, PM5_mod,PM2_sup, PP3_mod |
| SOD#18 | heterozygous for: g.33039603A>C, NM_000454.5(SOD1):c.272A>C, p.(Asp91Ala) | 4 | PM3_str, PM1_mod, PS3_sup, PM5_sup |
| SOD#19 | heterozygous for: g.33039603A>C, NM_000454.5(SOD1):c.272A>C, p.(Asp91Ala) | 4 | PM3_str, PM1_mod, PS3_sup, PM5_sup |
| SOD#20 | heterozygous for: NM_000454.5(SOD1):c.352C>G / p.(Leu118Val) | 4 | PS4_str, PM1_mod, PS3_sup, PM2_sup |
| SOD#21 | heterozygous for NM_000454.5(SOD1):c.131A>G/p.(His44Arg) | 4 | PS4_str, PM2_sup, PP3_mod |
| SOD#22 | heterozygous for NM_000454.5(SOD1):c.32G>C/p.(Gly11Ala) | 4 | PS4_str, PM2_sup, PP3_mod |
| SOD#23 | heterozygous for NM_000454.5(SOD1):c.32G>C/p.(Gly11Ala) | 4 | PS4_str, PM2_sup, PP3_mod |
| SOD#24 | heterozygous for NM_000454.5(SOD1):c.446T>C/p.(Val149Ala) | 3 | PM2_sup, PM5_mod, PP3_mod |
| SOD#25 | heterozygous for: NM_000454.5(SOD1):c.435G>C/p.(Leu145Phe) | 5 | PS4_str, PP1_sup, PS3_sup, PS1_str, PM2_sup, PP3_mod |
| SOD#26 | heterozygous for: NM_000454.5(SOD1):c.435G>C/p.(Leu145Phe) | 5 | PS4_str, PP1_sup, PS3_sup, PS1_str, PM2_sup, PP3_mod |
| SOD#27 | heterozygous for NM_000454.5(SOD1):c.435G>C/p.(Leu145Phe) | 5 | PS4_str, PP1_sup, PS3_sup, PS1_str, PM2_sup, PP3_mod |
| SOD#28 | homozygous for: NM_000454.5(SOD1):c.197A>G, p.(Asn66Ser) | 4 | PS4_str, PM2_sup, PP3_mod |
| SOD#29 | heterozygous for: NM_000454.5(SOD1):c.445G>A/p.(Val149Ile) | 4 | PS4_mod, PM2_sup, PM5_sup, PP3_sup, PS3_sup |
| SOD#30 | heterozygous for NM_000454.5(SOD1):c.358-10T>G | 4 | PS4_mod, PP1_mod, PM2_sup, PP3_sup |
| SOD#31 | heterozygous for: NM_000454.5(SOD1):c.435G>C/p.(Leu145Phe) | 5 | PS4_str, PP1_sup, PS3_sup, PS1_str, PM2_sup, PP3_mod |
| SOD#32 | heterozygous for: NM_000454.5(SOD1):c.400G>A/p.(Glu134Lys) | 4 | PS4_mod, PM2_sup, PM5_sup, PP3_mod |
| SOD#33 | homozygous for: g.33039603A>C, NM_000454.5(SOD1):c.272A>C, p.(Asp91Ala) | 4 | PM3_str, PM1_mod, PS3_sup, PM5_sup |
| SOD#34 | heterozygous for: NM_000454.5(SOD1):c.197A>G/Pp.(Asn66Ser) | 4 | PS4_str, PM2_sup, PP3_mod |
| SOD#35 | heterozygous | 4 | PM3_str, PM1_mod, PS3_sup, PM5_sup |
| SOD#36 | heterozygous for: NM_000454.5(SOD1):c.435G>T/p.(Leu145Phe) | 4 | PS4_mod, PS1_str, PM2_sup, PP3_mod |
| SOD#37 | heterozygous for: NM_000454.5(SOD1):c.269C>T, p.(Ala90Val) | 4 | PS4_mod, PP1_sup, PM5_sup, PP3_mod |
| SOD#38 | heterozygous for : NM_000454.5(SOD1):c.346C>G, p.(Arg116Gly) | 5 | PS4_str, PM1_mod, PM5_mod,PM2_sup, PP3_mod |
| SOD#39 | heterozygous for: NM_000454.5(SOD1):c.400_402del, p.(Glu134del) | 4 | PS4_mod, PM4_mod, PM5_sup, PM2_sup |
| SOD#40 | heterozygous for: NM_000454.5(SOD1):c.262G>A/p.(Val88Met) | 4 | PS4_mod, PM2_sup, PM5_sup, PP3_mod |
| SOD#41 | heterozygous for: NM_000454.5(SOD1):c.446T>G/p.(Val149Gly) | 4 | PS4_str, PS3_sup, PM2_sup, PM5_sup, PP3_mod |
| SOD#42 | herterozygous for: g.33039603A>C, NM_000454.5(SOD1):c.272A>C, p.(Asp91Ala) | 4 | PM3_str, PM1_mod, PS3_sup, PM5_sup |
| SOD#43 | homozygous for: g.33039603A>C, NM_000454.5(SOD1):c.272A>C, p.(Asp91Ala) | 4 | PM3_str, PM1_mod, PS3_sup, PM5_sup |
| SOD#44 | heterozygous for: NM_000454.5(SOD1):c.140A>G/ p.(His47Arg) | 5 | PS4_str, PP1_sup,PS3_mod, PM2_sup, PP3_mod |
| SOD#45 | heterozygous for: NM_000454.5(SOD1):c.125G>A/p.(Gly42Asp) | 5 | PS4_str, PS3_sup, PM2_sup, PM5_mod, PP3_mod, (PP1_mod) |
| SOD#46 | heterozygous for: NM_000454.5(SOD1):c.146A>C(p.(His49Pro) | 3 | PM2_sup, PM5_sup, PP3_mod |
| SOD#47 | heterozygous for: NM_000454.5(SOD1):c.346C>G/p.(Arg116Gly), rs1301635320 | 5 | PS4_str, PM1_mod, PM5_mod,PM2_sup, PP3_mod |
| SOD#48 | heterozygous for NM_000454.5(SOD1):c.341T>C, p.(Ile114Thr) | 4 | PS4_mod, PM1_mod, PS3_sup, PP3_mod |
| SOD#49 | heterozygous for NM_000454.5(SOD1):c.131A>G/ p.(His44Arg) | 4 | PS4_str, PM2_sup, PP3_mod |
| SOD#50 | heterozygous for: g.33039603A>C, NM_000454.5(SOD1):c.272A>C, p.(Asp91Ala) | 4 | PM3_str, PM1_mod, PS3_sup, PM5_sup |
| SOD#51 | heterozygous for: for: g.33039603A>C, NM_000454.5(SOD1):c.272A>C, p.(Asp91Ala) | 4 | PM3_str, PM1_mod, PS3_sup, PM5_sup |
| SOD#52 | homozygous for: NM_000454.5(SOD1):c.412A>G/p.(Thr138Ala) | 4 | PS4_mod, PM2_sup, PM5_sup, PP3_mod |
| SOD#53 | heterozygous for splice donor mutation: NM_000454.5(SOD1):c.73-1G>A | 4 | PVS1_vstr, PM2_sup |
| SOD#54 | heterozygous for NM_000454.5(SOD1):c.435G>C/p.(Leu145Phe) | 5 | PS4_str, PP1_sup, PS3_sup, PS1_str, PM2_sup, PP3_mod |
| TARDBP#1 | heterozygous for g.11082521A>G, cDNA Level: NM_007375.4(TARDBP):c.1055A>G, p.(Asn352Ser) | 4 | PS4_str, PS3_sup, PM1_mod, PM2_sup |
| TARDBP#2 | heterozygous for g.11082521A>G, NM_007375.4(TARDBP):c.1055A>G, p.(Asn352Ser) | 4 | PS4_str, PS3_sup, PM1_mod, PM2_sup |
| TARDBP#3 | heterozygous for g.11082509G>T, NM_007375.4(TARDBP):c.1043G>T, p.(Gly348Val) | 4 | PS4_str, PM1_mod, PM2_sup, PM5_mod |
| TARDBP#4 | heterozygous for g.11082521A>G/NM_007375.4(TARDBP):c.1055A>G, p.(Asn352Ser) | 4 | PS4_str, PS3_sup, PM1_mod, PP2_sup, PM2_sup |
| TARDBP#5 | heterozygous for NM_007375.4(TARDBP):c.1055A>G/p.(Asn352Ser) | 4 | PS4_str, PS3_sup, PM1_mod, PM2_sup |
| TARDBP#6 | heterozygous for: NM_007375.4(TARDBP):c.1169A>G/p.(Asn390Ser) | 4 | PS4_sup, PM1_mod, PM5_sup, PS3_mod |
| TARDBP#7 | heterozygous for: NM_007375.4(TARDBP):c.1055A>G/p.(Asn352Ser) | 4 | PS4_str, PS3_sup, PM1_mod, PM2_sup |
| TARDBP#8 | heterozygous for missense variant: NM_007375.4(TARDBP):c.1147A>G/ p.(Ile383Val) | 4 | PS4_str, PM1_mod, PM2_sup |
| TARDBP#9 | heterozygous for: NM_007375.4(TARDBP):c.1147A>G/p.(Ile383Val) | 4 | PS4_str, PM1_mod, PM2_sup |
| TARDBP#10 | heterozygot for:NM_007375.4(TARDBP):c.1169A>G/p.(Asn390Ser) | 4 | PS4_sup, PM1_mod, PM5_sup, PS3_mod |
| TARDBP#11 | heterozygot for: NM_007375.4(TARDBP):c.1055A>G, p.(Asn352Ser) | 4 | PS4_str, PS3_sup, PM1_mod, PM2_sup |
| TARDBP#12 | heterozygous for TARDBP:c.714+9C>T. | 3 | PM2_sup (In-silico-tools do not predict aberrant splicing) |
| TARDBP#13 | heterozygous for: NM_007375.4(TARDBP):c.859G>A/p.(Gly287Ser) | 3 | PS4_sup, PS3_mod, PM1_mod |
| TARDBP#14 | heterozygous for: TARDBP:c.875G>T/p.(Ser292Ile) | 3 | PM1_mod, PM2_sup |
| TARDBP#15 | heterozygous for NM_007375.4(TARDBP):c.943G>A/p.Ala315Thr, | 5 | PS4_str, PP1_str, PM1_mod, PM2_sup |
| TARDBP#16 | heterozygous for: NM_007375.4(TARDBP):c.1055A>G/p.(Asn352Ser) | 4 | PS4_str, PS3_sup, PM1_mod, PM2_sup |
| TARDBP#17 | heterozygous for: NM_007375.4(TARDBP):c.931A>G/p.(Met311Val) | 4 | PS4_mod, PM1_mod, PM2_sup, PS3_sup |
| TARDBP#18 | heterozygous for: NM_007375.4(TARDBP):c.943G>A/p.(Ala315Thr) | 5 | PS4_str, PP1_str, PM1_mod, PM2_sup |
| TARDBP#19 | heterozygous for: NM_007375.4(TARDBP):c.1055A>G, p.(Asn352Ser) | 4 | PS4_str, PS3_sup, PM1_mod, PM2_sup |
| TARDBP#20 | heterozygous for: NM_007375.4(TARDBP):c.1055A>G, p.(Asn352Ser) | 4 | PS4_str, PS3_sup, PM1_mod, PM2_sup |
| TARDBP#21 | heterozygous for: NM_007375.4(TARDBP):c.859G>A/p.(Gly287Ser) | 3 | PS4_sup, PS3_mod, PM1_mod |
| TARDBP#22 | heterozygous for NM_007375.4(TARDBP):c.869G>C/p.(Gly290Ala) | 3 | PS4_sup, PP1_sup, PM1_mod, PM2_sup |
| TARDBP#23 | heterozygous for: NM_007375.4(TARDBP):c.920T>C/p.(Met307Thr) | 3 | PM1_mod, PM2_sup |
| TARDBP#24 | heterozygous for NM_007375.4(TARDBP):c.1055A>G/p.(Asn352Ser), | 4 | PS4_str, PS3_sup, PM1_mod, PM2_sup |
| TARDBP#25 | heterozygous for NM_007375.4(TARDBP):c.1055A>G/p.(Asn352Ser) | 4 | PS4_str, PS3_sup, PM1_mod, PM2_sup |
| TARDBP#26 | heterozygous for: NM_007375.4(TARDBP):c.1055A>G/p.(Asn352Ser) | 4 | PS4_str, PS3_sup, PM1_mod, PM2_sup |
| TARDBP#27 | heterozygous for: NM_007375.4(TARDBP):c.1055A>G/p.(Asn352Ser) | 4 | PS4_str, PS3_sup, PM1_mod, PM2_sup |
| FUS#1 | heterozygous: g.31202284G>A, NM_004960.4(FUS):c.1394G>A, p.(Gly465Glu) | 3 | "-" |
| FUS#2 | heterozygous for: g.31202330del, NM_004960.4(FUS):c.1440del, p.(Arg481Glufs*48) | 3 | PVS1_str, PM2_sup |
| FUS#3 | heterozygot for: g.31202419A>G, NM_004960.4(FUS):c.1529A>G, p.(Lys510Arg) | 4 | PS4_str, PP1_sup, PM2_sup, PP3_mod |
| FUS#4 | heterozygous for:NM_004960.4(FUS):c.197A>G/p.(Tyr66Cys) | 3 | PP3_sup |
| FUS#5 | heterozygous for: NM_004960.4(FUS):c.1530G>T/p.(Lys510Asn) | 3 | PM2_sup, PM5_sup, PP3_sup |
| FUS#6 | heterozygot für: NM_004960.4(FUS):c.1562G>A/p.(Arg521His) | 5 | PS4_str, PS3_sup, PM2_sup, PM5_mod, PM1_mod |
| FUS#7 | heterozygous for NM_004960.4(FUS):c.1562G>A/p.(Arg521His) | 5 | PS4_str, PS3_sup, PM2_sup, PM5_mod, PM1_mod |
| FUS#8 | heterozygous for: NM_004960.4(FUS):c.41A>G/p.(Tyr14Cys) | 3 | PM2_sup |
| FUS#9 | heterozygous for NM_004960.4(FUS):c.716A>G/p.(Tyr239Cys) | 3 | PS4_sup |
| FUS#10 | heterozygous for NM_004960.4(FUS):c.1453C>T/p.(Arg485Trp) | 3 | BS1_str |
| FUS#11 | heterozygous for: NM_004960.4(FUS):c.1559_1561delinsTTT/ in-frame p.(Asp520_Arg521delinsValCys). | 5 | R521C: PS4_str, PM2_sup, PM5_mod, PP3_sup (Class 5); D520V: PM2_sup (Class 3) |
| FUS#12 | Heterozygous for: NM_004960.4(FUS):c.317C>T/p.(Pro106Leu) | 3 | PS4_sup |
| FUS#13 | heterozygous for variant of uncertain significance: NM_004960.4(FUS):c.52C>T/p.(Pro18Ser) | 3 | BS1_str |
| FUS#14 | heterozygous for: NM_004960.4(FUS):c.1555C>G/p.(Gln519Glu) | 3 | PS4_sup, PM2_sup, PM1_mod |
| FUS#15 | heterozygous for: NM_004960.4(FUS):c.1562G>A/p.(Arg521His) | 5 | PS4_str, PS3_sup, PM2_sup, PM5_mod, PM1_mod |
| FUS#16 | heterozygous for NM_004960.4(FUS):c.646C>T/p.(Arg216Cys) | 3 | PP3_sup |
| FUS#17 | heterozygous for: NM_004960.4(FUS):c.451C>T, p.(Pro151Ser) | 3 | PS4_sup |
| FUS#18 | heterozygous for : NM_004960.4(FUS):c.1400A>T/p.(Asn467Ile). | 3 | PM2_sup, BP4_sup |
| FUS#19 | heterozygous for: NM_004960.4(FUS):c.425G>A/p.(Ser142Asn) | 3 | PM2_sup |

**Supplementary Table 1. Genetic Variant Analysis and Classification.**

Detailed information about the patients with genetic variants of the *SOD1*, *FUS* and *TARDBP* genes according to the ACMG guidelines and modified based on the recommendations of the ClinGen Variant Classification Guidance
