# Supplementary material for: Motor phenotypes and neurofilament light chain in genetic amyotrophic lateral sclerosis—results from a multicenter screening program
Source: J Neurol. 2025 Dec 12;273(1):22. doi: 10.1007/s00415-025-13555-6 (PMC12700978; doi:10.1007/s00415-025-13555-6)
Supplement: Supplementary file 2 — Supplementary file2 (DOCX 63 KB) [file 415_2025_13555_MOESM2_ESM.docx]

| *Patient* | *Onset* | | *Propagation pattern* | | *Motor neuron involvement* | | *sNfL (pg/ml)* | *ALSPR* |
| --- | --- | --- | --- | --- | --- | --- | --- | --- |
|  | ***region*** | ***O*** | ***type*** | ***P*** | ***type*** | ***M*** |  |  |
|  |  |  |  |  |  |  |  |  |
| C9orf72 #1 | head | O1 | late | PL | classic | M0 | 241,9 | 0,25 |
| C9orf72 #2 | head | O1 | early | PE | classic | M0 | 229,7 | 4,6 |
| C9orf72 #3 | head | O1 | not specified | PX | UMNp | M1d | 198,4 | 1,5 |
| C9orf72 #4 | arm | O2 | early | PE | classic | M0 | 86,5 | 0,37 |
| C9orf72 #5 | leg | O4 | early | PE | classic | M0 | 71 | 0,9 |
| C9orf72 #6 | leg | O4 | early | PE | classic | M0 | 194,9 | 0,43 |
| C9orf72 #7 | leg | O4 | early | PE | classic | M0 | 71,3 | 0,88 |
| C9orf72 #8 | head | O1 | late | PL | classic | M0 | 171,3 | 1,08 |
| C9orf72 #9 | head | O1 | late | PL | classic | M0 | 63,3 | 0,38 |
| C9orf72 #10 | leg | O4 | early | PE | UMNp | M1d | 58,3 | 0,18 |
| C9orf72 #11 | head | O1 | not specified | PX | classic | M0 | 63,1 | 0,21 |
| C9orf72 #12 | arm | O2 | early | PE | classic | M0 | 135,9 | 0,67 |
| C9orf72 #13 | leg | O4 | early | PE | UMNp | M1d | 63,6 | 0,42 |
| C9orf72 #14 | leg | O4 | early | PE | UMNp | M1d | 4,1 | 0,5 |
| C9orf72 #15 | head | O1 | early | PE | classic | M0 | 97,5 | 1,67 |
| C9orf72 #16 | arm | O2 | early | PE | classic | M0 | n/o | 0,57 |
| C9orf72 #17 | head | O1 | early | PE | classic | M0 | 222 | 0,8 |
| C9orf72 #18 | leg | O4 | early | PE | UMNp | M1d | 65 | 1,17 |
| C9orf72 #19 | leg | O4 | early | PE | classic | M0 | 54,9 | 0,95 |
| C9orf72 #20 | arm | O2 | early | PE | LMNp | M2d | 23,6 | 0,05 |
| C9orf72 #21 | arm | O2 | early | PE | classic | M0 | 76 | 0,3 |
| C9orf72 #22 | leg | O4 | early | PE | LMNp | M2d | 50,2 | 0,75 |
| C9orf72 #23 | head | O1 | early | PE | classic | M0 | 72,3 | 0,33 |
| C9orf72 #24 | leg | O4 | early | PE | classic | M0 | 92,7 | 0,62 |
| C9orf72 #25 | head | O1 | not specified | PE | UMNp | M1d | 182 | 0,86 |
| C9orf72 #26 | head | O1 | early | PE | UMNp | M1d | 175 | 2,67 |
| C9orf72 #27 | leg | O4 | early | PE | LMNp | M2d | 68,4 | 0,63 |
| C9orf72 #28 | head | O1 | early | PE | classic | M0 | 74,5 | 0,33 |
| C9orf72 #29 | arm | O2 | early | PE | classic | M0 | 49,5 | 1,36 |
| C9orf72 #30 | arm | O2 | late | PL | LMNp | M2d | 20,9 | 0,03 |
| C9orf72 #31 | head | O1 | not specified | PX | UMNp | M1d | 56,4 | 0,55 |
| C9orf72 #32 | arm | O2 | early | PE | classic | M0 | 40,3 | 0,21 |
| C9orf72 #33 | leg | O4 | early | PE | classic | M0 | 79,7 | 0,21 |
| C9orf72 #34 | head | O1 | late | PL | classic | M0 | 118,2 | 0,75 |
| C9orf72 #35 | leg | O4 | late | PL | classic | M0 | 85,5 | 0,61 |
| C9orf72 #36 | arm | O2 | early | PE | classic | M0 | 126 | 0,71 |
| C9orf72 #37 | head | O1 | late | PL | classic | M0 | 85,8 | 0,6 |
| C9orf72 #38 | arm | O2 | early | PE | LMNp | M2d | 35,7 | 0,6 |
| C9orf72 #39 | head | O1 | early | PE | classic | M0 | 54,5 | 0,67 |
| C9orf72 #40 | head | O1 | early | PE | classic | M0 | 64,2 | 2,2 |
| C9orf72 #41 | head | O1 | early | PE | classic | M0 | 87,5 | 0,38 |
| C9orf72 #42 | head | O1 | early | PE | UMNp | M1d | 142,8 | n/o |
| C9orf72 #43 | arm | O2 | early | PE | LMNp | M2d | 114 | 0,32 |
| C9orf72 #44 | leg | O4 | early | PE | classic | M0 | n/o | 2,13 |
| C9orf72 #45 | head | O1 | late | PL | classic | M0 | 123 | 0,74 |
| C9orf72 #46 | head | O1 | not specified | PX | classic | M0 | 140,1 | 0,21 |
| C9orf72 #47 | arm | O2 | early | PE | classic | M0 | 49,1 | 0,33 |
| C9orf72 #48 | leg | O4 | early | PE | classic | M0 | 55,5 | 0,33 |
| C9orf72 #49 | head | O1 | early | PE | classic | M0 | 149,1 | 0,83 |
| C9orf72 #50 | arm | O2 | early | PE | classic | M0 | 34,4 | 0,45 |
| C9orf72 #51 | head | O1 | early | PE | classic | M0 | 199 | 0,67 |
| C9orf72 #52 | leg | O4 | early | PE | UMNp | M1d | 117,5 | 0,42 |
| C9orf72 #53 | leg | O4 | early | PE | classic | M0 | 160 | 0,93 |
| C9orf72 #54 | leg | O4 | early | PE | classic | M0 | 119 | 0,69 |
| C9orf72 #55 | head | O1 | late | PL | UMNp | M1d | 25 | 0,08 |
| C9orf72 #56 | limb (not further classified) | OX | early | PE | UMNp initial | M1d | 127,6 | 0,93 |
| C9orf72 #57 | head | O1 | not specified | PX | classic | M0 | 100 | 1 |
| C9orf72 #58 | head | O1 | not specified | PX | classic | M0 | 125 | 0,87 |
| C9orf72 #59 | leg | O4 | early | PE | classic | M0 | 123 | 2,14 |
| C9orf72 #60 | head | O1 | early | PE | classic | M0 | 168 | 0,51 |
| C9orf72 #61 | leg | O4 | early | PE | LMNp | M2d | 76,6 | 0,46 |
| C9orf72 #62 | leg | O4 | early | PE | LMNp | M2d | 91 | 0,12 |
| C9orf72 #63 | head | O1 | early | PE | classic | M0 | 95,1 | 0,33 |
| C9orf72 #64 | head | O1 | early | PE | classic | M0 | 92,6 | 1,63 |
| C9orf72 #65 | arm | O2 | early | PE | classic | M0 | 133 | 0,45 |
| C9orf72 #66 | arm | O2 | late | PL | classic | M0 | 42,9 | 0,14 |
| C9orf72 #67 | head | O1 | early | PE | classic | M0 | 180 | 0,89 |
| C9orf72 #68 | arm | O2 | early | PE | classic | M0 | 42 | 0,58 |
| C9orf72 #69 | arm | O2 | late | PL | LMNp | M2d | 60,3 | 0,34 |
| C9orf72 #70 | leg | O4 | early | PE | classic | M0 | 84,8 | 0,43 |
| C9orf72 #71 | head | O1 | early | PE | classic | M0 | 119 | 0,14 |
| C9orf72 #72 | leg | O4 | early | PE | classic | M0 | 138 | 0,45 |
| C9orf72 #73 | arm | O2 | early | PE | classic | M0 | n/o | 1,17 |
| C9orf72 #74 | arm | O2 | early | PE | classic | M0 | 153 | 1,67 |
| C9orf72 #75 | head | O1 | early | PE | classic | M0 | 104 | 2 |
| C9orf72 #76 | leg | O4 | early | PE | UMNp | M1d | 178 | 1,62 |
| C9orf72 #77 | head | O1 | late | PL | classic | M0 | 436 | 1,13 |
| C9orf72 #78 | head | O1 | early | PE | classic | M0 | 152 | 0,35 |
| C9orf72 #79 | arm | O2 | early | PE | classic | M0 | 108 | 0,6 |
| C9orf72 #80 | head | O1 | early | PE | classic | M0 | 270 | 2,14 |
| C9orf72 #81 | arm | O2 | early | PE | classic | M0 | 149 | 1 |
| C9orf72 #82 | head | O1 | not specified | PX | classic | M0 | 87,7 | 0,9 |
| C9orf72 #83 | limb (not further classified) | OX | early | PE | classic | M0 | 184 | 1,3 |
| C9orf72 #84 | head | O1 | not specified | PX | classic | M0 | 103 | 0,86 |
| C9orf72 #85 | arm | O2 | early | PE | dissociated | M3 | 114 | 1,18 |
| C9orf72 #86 | head | O1 | late | PL | classic | M0 | 49 | 1,43 |
| C9orf72 #87 | leg | O4 | early | PE | classic | M0 | 229 | 2,75 |
| C9orf72 #88 | head | O1 | early | PE | classic | M0 | 141 | 2,83 |
| C9orf72 #89 | arm | O2 | early | PE | classic | M0 | 5,5 | n/o |
| C9orf72 #90 | arm | O2 | early | PE | classic | M0 | 70,2 | 0,03 |
| C9orf72 #91 | head | O1 | early | PE | classic | M0 | 352 | 0,83 |
| C9orf72 #92 | arm | O2 | early | PE | classic | M0 | 104 | 0,33 |
| C9orf72 #93 | arm | O2 | early | PE | classic | M0 | 75 | 3,67 |
| C9orf72 #94 | head | O1 | early | PE | classic | M0 | 82,2 | 0,83 |
| C9orf72 #95 | leg | O4 | early | PE | classic | M0 | 123 | 0,88 |
| C9orf72 #96 | head | O1 | early | PE | classic | M0 | 62,4 | 1,38 |
| C9orf72 #97 | head | O1 | early | PE | classic | M0 | n/o | 2,11 |
| C9orf72 #98 | leg | O4 | early | PE | classic | M0 | n/o | 1,38 |
| C9orf72 #99 | head | O1 | early | PE | classic | M0 | 82,4 | 1 |
| C9orf72 #100 | arm | O2 | late | PL | classic | M0 | n/o | 0,15 |
| C9orf72 #101 | head | O1 | not specified | PX | UMNp | M1d | 109 | 0,74 |
| C9orf72 #102 | leg | O4 | early | PE | classic | M0 | 105 | 0,64 |
| C9orf72 #103 | head | O1 | early | PE | classic | M0 | 61,5 | 0,08 |
| C9orf72 #104 | leg | O4 | early | PE | classic | M0 | 269 | 2,75 |
| C9orf72 #105 | leg | O4 | early | PE | LMNp | M2d | 83,4 | 0,76 |
| C9orf72 #106 | head | O1 | not specified | PX | classic | M0 | n/o | 0,3 |
| C9orf72 #107 | head | O1 | not specified | PX | classic | M0 | 272,6 | 0,42 |
| C9orf72 #108 | head | O1 | early | PE | classic | M0 | 92,7 | 0,92 |
| C9orf72 #109 | leg | O4 | early | PE | classic | M0 | 175 | 1,4 |
| C9orf72 #110 | leg | O4 | early | PE | classic | M0 | 353 | 3,63 |
| C9orf72 #111 | leg | O4 | early | PE | UMNp | M1d | 71,4 | 1,2 |
| C9orf72 #112 | leg | O4 | early | PE | UMNp | M1d | 193 | 2,71 |
| C9orf72 #113 | head | O1 | not specified | PX | classic | M0 | 74 | 0,87 |
| C9orf72 #114 | head | O1 | early | PE | UMNp | M1d | 88,9 | 1,91 |
| C9orf72 #115 | leg | O4 | early | PE | dissociated | M3 | 39,3 | 0,5 |
| C9orf72 #116 | head | O1 | early | PE | classic | M0 | 80,7 | 0,57 |
| C9orf72 #117 | leg | O4 | early | PE | classic | M0 | 43,5 | 0,31 |
| C9orf72 #118 | leg | O4 | early | PE | classic | M0 | 84,3 | 0,14 |
| C9orf72 #119 | arm | O2 | early | PE | dissociated | M3 | 67,5 | 0,13 |
| C9orf72 #120 | arm | O2 | early | PE | LMNp | M2d | 84,2 | 1,18 |
| C9orf72 #121 | leg | O4 | early | PE | classic | M0 | 141 | 0,63 |
| C9orf72 #122 | leg | O4 | early | PE | classic | M0 | 205 | 2,88 |
| C9orf72 #123 | not specified | OX | not specified | PX | classic | M0 | n/o | n/o |
| C9orf72 #124 | not specified | OX | not specified | PX | classic | M0 | n/o | n/o |
| C9orf72 #125 | not specified | OX | not specified | PX | UMNp | M1d | n/o | n/o |
| SOD1#1 | arm | O2 | early | PE | LMNp | M2d | 16,3 | 0,06 |
| SOD1#2 | leg | O4 | early | PE | classic | M0 | 302 | 0,67 |
| SOD1#3 | leg | O4 | early | PE | LMNp | M2d | 37,4 | 0,42 |
| SOD1#4 | leg | O4 | early | PE | LMNp | M2d | 100,7 | 1,29 |
| SOD1#5 | leg | O4 | early | PE | classic | M0 | 50,8 | 0,24 |
| SOD1#6 | arm | O2 | late | PL | LMNp | M2d | 87,2 | 0,05 |
| SOD1#7 | arm | O2 | early | PE | classic | M0 | 90,3 | 0,81 |
| SOD1#8 | arm | O2 | late | PL | LMNp | M2d | 33,8 | 0,29 |
| SOD1#9 | leg | O4 | early | PE | classic | M0 | 66,9 | 0,7 |
| SOD1#10 | arm | O2 | early | PE | classic | M0 | 282 | 1,2 |
| SOD1#11 | leg | O4 | early | PE | classic | M0 | 69,6 | 0,22 |
| SOD1#12 | trunk | O3 | early | PE | LMNp | M2d | 107,1 | 2,63 |
| SOD1#13 | arm | O2 | early | PE | classic | M0 | n/o | 0,16 |
| SOD1#14 | leg | O4 | late | PL | classic | M0 | 61,9 | 0,29 |
| SOD1#15 | leg | O4 | late | PL | LMNp | M2d | 80,2 | 0,46 |
| SOD1#16 | leg | O4 | late | PL | LMNp | M2d | n/o | 0,09 |
| SOD1#17 | leg | O4 | early | PE | classic | M0 | 76,9 | 0,37 |
| SOD1#18 | leg | O4 | early | PE | classic | M0 | 120,1 | 0,29 |
| SOD1#19 | leg | O4 | early | PE | classic | M0 | 68 | 0,4 |
| SOD1#20 | leg | O4 | late | PL | LMNp | M2d | 27,5 | 1,1 |
| SOD1#21 | arm | O2 | early | PE | classic | M0 | 96,4 | n/o |
| SOD1#22 | leg | O4 | early | PE | classic | M0 | 15 | 0,61 |
| SOD1#23 | leg | O4 | early | PE | classic | M0 | 107 | 1,5 |
| SOD1#24 | leg | O4 | early | PE | classic | M0 | 33,9 | 0,42 |
| SOD1#25 | leg | O4 | late | PL | LMNp | M2d | n/o | 0,11 |
| SOD1#26 | leg | O4 | early | PE | UMNp | M1d | 21,5 | 1,2 |
| SOD1#27 | leg | O4 | late | PL | LMNp | M2d | n/o | 0,18 |
| SOD1#28 | leg | O4 | late | PL | LMNp | M2d | 12 | 0,11 |
| SOD1#29 | head | O1 | late | PL | LMNp | M2d | 57,7 | 1,67 |
| SOD1#30 | leg | O4 | late | PL | LMNp | M2d | 41,9 | 0,1 |
| SOD1#31 | arm | O2 | late | PL | classic | M0 | 15,5 | 0,05 |
| SOD1#32 | leg | O4 | early | PE | LMNp | M2d | 161 | 1,17 |
| SOD1#33 | leg | O4 | early | PE | LMNp | M2d | 167 | n/o |
| SOD1#34 | arm | O2 | early | PE | classic | M0 | 91,7 | 2,5 |
| SOD1#35 | leg | O4 | late | PL | LMNp | M2d | 52,7 | 0,25 |
| SOD1#36 | leg | O4 | early | PE | classic | M0 | 71,2 | 1,42 |
| SOD1#37 | leg | O4 | early | PE | classic | M0 | n/o | 0,42 |
| SOD1#38 | leg | O4 | early | PE | UMNp | M1d | 83,8 | 0,35 |
| SOD1#39 | leg | O4 | early | PE | classic | M0 | 53,9 | 0,09 |
| SOD1#40 | leg | O4 | early | PE | classic | M0 | 39,5 | 0,07 |
| SOD1#41 | leg | O4 | early | PE | LMNp | M2d | 98,5 | 0,34 |
| SOD1#42 | leg | O4 | early | PE | classic | M0 | 118,7 | 0,27 |
| SOD1#43 | leg | O4 | early | PE | UMNp | M1d | 15 | 0,1 |
| SOD1#44 | arm | O2 | early | PE | classic | M0 | 36,5 | 0,59 |
| SOD1#45 | arm | O2 | early | PE | classic | M0 | 36,5 | 0,15 |
| SOD1#46 | leg | O4 | early | PE | classic | M0 | 60,4 | 0,57 |
| SOD1#47 | leg | O4 | late | PL | LMNp | M2d | 30,5 | 0,2 |
| SOD1#48 | leg | O4 | early | PE | UMNp | M1d | 37,2 | 0,14 |
| SOD1#49 | leg | O4 | early | PE | classic | M0 | 10,1 | 0,33 |
| SOD1#50 | leg | O4 | early | PE | classic | M0 | 154 | 0,4 |
| SOD1#51 | arm | O2 | late | PL | LMNp | M2d | 7,5 | 0,33 |
| TARDBP#1 | arm | O2 | late | PL | LMNp | M2d | 23,3 | 0,24 |
| TARDBP#2 | arm | O2 | early | PE | dissociated | M3 | 29,6 | 0,67 |
| TARDBP#3 | arm | O2 | early | PE | dissociated | M3 | 35,5 | 0,22 |
| TARDBP#4 | arm | O2 | early | PE | classic | M0 | 39,4 | 2 |
| TARDBP#5 | arm | O2 | early | PE | classic | M0 | 46 | 0,33 |
| TARDBP#6 | leg | O4 | early | PE | UMNp | M1d | 21 | 0,21 |
| TARDBP#7 | arm | O2 | early | PE | LMNp | M2d | 28,4 | 0,53 |
| TARDBP#8 | leg | O4 | early | PE | classic | M0 | 48,4 | 0,22 |
| TARDBP#9 | leg | O4 | early | PE | classic | M0 | 49,3 | 0,37 |
| TARDBP#10 | arm | O2 | early | PE | LMNp | M2d | 23,4 | 0,42 |
| TARDBP#11 | leg | O4 | early | PE | classic | M0 | n/o | 0,39 |
| TARDBP#12 | leg | O4 | early | PE | classic | M0 | 25,5 | 0,7 |
| TARDBP#13 | leg | O4 | early | PE | LMNp | M2d | 16 | 0,29 |
| TARDBP#14 | arm | O2 | early | PE | classic | M0 | 38,5 | 0,37 |
| TARDBP#15 | leg | O4 | early | PE | classic | M0 | 149 | 2,2 |
| TARDBP#16 | arm | O2 | late | PL | LMNp | M2d | 24,5 | 0,12 |
| TARDBP#17 | arm | O2 | early | PE | classic | M0 | 9,7 | 0,42 |
| TARDBP#18 | arm | O2 | early | PE | UMNp | M1d | 85,5 | 0,39 |
| TARDBP#19 | arm | O2 | early | PE | classic | M0 | 42 | 0,25 |
| TARDBP#20 | arm | O2 | early | PE | dissociated | M3 | 35 | 0,56 |
| TARDBP#21 | head | O1 | early | PE | classic | M0 | 31,8 | 0,62 |
| TARDBP#22 | arm | O2 | late | PL | LMNp | M2d | 16,5 | 0,16 |
| TARDBP#23 | leg | O4 | late | PL | LMNp | M2d | n/o | n/o |
| TARDBP#24 | leg | O4 | early | PE | dissociated | M3 | 65 | n/o |
| FUS1#1 | leg | O4 | early | PE | LMNp | M2d | 59,8 | 1,68 |
| FUS1#2 | arm | O2 | late | PL | LMNp | M2d | 62,5 | 0,32 |
| FUS1#3 | limb (not further classified) | OX | early | PE | dissociated | M3 | 70,2 | 0,4 |
| FUS1#4 | arm | O2 | early | PE | LMNp | M2d | 100 | 0,25 |
| FUS1#5 | leg | O4 | late | PL | LMNp | M2d | 71,5 | 0,3 |
| FUS1#6 | leg | O4 | late | PL | LMNp | M2d | 54,4 | 3 |
| FUS1#7 | arm | O2 | late | PL | LMNp | M2d | 33 | 1,5 |
| FUS1#8 | head | O1 | early | PE | classic | M0 | 52,5 | 0,84 |
| FUS1#9 | leg | O4 | late | PL | LMNp | M2d | 31,3 | 0,67 |
| FUS1#10 | arm | O2 | early | PE | LMNp | M2d | 70,9 | 0,38 |
| FUS1#11 | arm | O2 | late | PL | LMNp | M2d | 105 | 0,44 |
| FUS1#12 | arm | O2 | late | PL | LMNp | M2d | 63 | 0,71 |
| FUS1#13 | head | O1 | early | PE | classic | M0 | 58,9 | 0,46 |
| FUS1#14 | trunk | O3 | early | PE | LMNp | M2d | 12 | 0,12 |
| FUS1#15 | leg | O4 | early | PE | classic | M0 | 121 | 0,69 |
| FUS1#16 | arm | O2 | late | PL | LMNp | M2d | 26,9 | 0,15 |
| FUS1#17 | leg | O4 | early | PE | classic | M0 | n/o | n/o |

**Supplementary Table 2. Distribution of OPM phenotypes by genotype.**

Abbreviations: *ALSPR*, ALS progression rate; *LMN*, lower motor neuron; *UMN*, upper motor neuron; *sNfL*, serum neurofilament light chain; *M0*, balanced UMN and LMN dysfunction; *M1d,* dominant UMN dysfunction; *M2d*, dominant LMN dysfunction; *M3*, dissociated motor neuron dysfunction
