# Supplementary material for: Motor phenotypes and neurofilament light chain in genetic amyotrophic lateral sclerosis—results from a multicenter screening program
Source: J Neurol. 2025 Dec 12;273(1):22. doi: 10.1007/s00415-025-13555-6 (PMC12700978; doi:10.1007/s00415-025-13555-6)
Supplement: Supplementary file 3 — Supplementary file3 (DOCX 17 KB) [file 415_2025_13555_MOESM3_ESM.docx]

|  | ***sNfL (pg/ml)*** | ***ALSPR*** | ***log NfL (index)*** |
| --- | --- | --- | --- |
|  |  |  |  |
| **Onset** |  |  |  |
| O1 (head) | 126.3 ± 80.46 | 1.0 ± 0.82 | 4.98 ± 0.87 |
| O2 (arm) | 64.36 ± 48.14 | 0.59 ± 0.64 | 4.94 ± 1.02 |
| O4 (leg) | 89.96 ± 66.01 | 0.78 ± 0.76 | 4.85 ± 0.86 |
|  |  |  |  |
| **Propagation** |  |  |  |
| PE (early) | 95.19 ± 66.41 | 0.87 ± 0.81 | 4.86 ± 0.9 |
| PL (late) | 70.65 ± 78.41 | 0.51 ± 0.59 | 5.0 ± 1.04 |
|  |  |  |  |
| **Degree of upper and lower motor neuron dysfunction** | | | |
| M0 (Balanced UMN and LMN) | 108.2 ± 76.16 | 0.87 ± 0.81 | 4.99 ± 0.88 |
| M2d (Dominant LMN) | 57.6 ± 37.98 | 0.57 ± 0.64 | 4.8 ± 1.0 |
| M1d (Dominant UMN) | 92.17 ± 61.92 | 0.9 ± 0.78 | 4.66 ±0.93 |

**Supplementary Table 3. Cross-mutation analysis of sNfL, ALSPR, and log NfL(index) across OPM subgroups**

Serum neurofilament light chain (NfL), ALS progression rate (ALSPR), and log-transformed NfL-index across motor phenotype determinants (onset region, propagation pattern, and degree of motor neuron involvement). Data are presented as mean ± standard deviation.
